# The inhibition of lactate/Gpr81 signalling driven by moderate-intensity continuous training protects against osteoarthritis

**DOI:** 10.1038/s12276-026-01819-8

**Published:** 2026-09-03

**Authors:** Peng Sun, Yilan Guo, Aochuan Xue, Jingting Ni, Yichen Ren, Yan Sun, Xingyu Qian, Xiaoliu Li, Yu You, Ying Zhao, Lijun Tang, Shimin Zhu, Yuqiang Li, Liang He

**Affiliations:** 1 The key Laboratory of Adolescent Health Assessment and Exercise Intervention of the Ministry of Education, East China Normal University, Shanghai, 200241 P. R. China; 2 Department of Veterinary Medicine, Shandong Vocational Animal Science and Veterinary College, Shandong, 261071 P. R. China; 3 Department of Rehabilitation, Minhang Hospital, Fudan University, Shanghai, 201199 P. R. China; 4 Shanghai Key Laboratory of Regulatory Biology, Institute of Biomedical Sciences and School of Life Sciences, East China Normal University, Shanghai, 200241 P. R. China; 5 Physical Education Department, Shanghai Jiao Tong University, Shanghai, 200042 P. R. China; 6 College of Physical Education, Shanghai Normal University, Shanghai, 200234 P. R. China; 7 Department of Orthopedics, Shanghai Key Laboratory for Prevention and Treatment of Bone and Joint Diseases, Shanghai Institute of Traumatology and Orthopedics, Ruijin Hospital, Shanghai Jiaotong University School of Medicine, Shanghai, 200245 P. R. China

**Keywords:** Metabolic bone disease, Extracellular signalling molecules

## Abstract

Osteoarthritis (OA) is among the most prevalent chronic diseases worldwide. However, currently employed pharmacological therapies for OA are associated with various reported side effects. Exercise therapy represents a potentially safer and more effective strategy for OA treatment, although its underlying mechanism remains unclear. Here, we demonstrate that compared with high-intensity continuous training, moderate-intensity continuous training (MICT) has greater therapeutic potential for OA. Notably, these beneficial effects of MICT were abrogated following gut microbiota depletion. Moreover, transplantation of the gut microbiota from MICT-treated mice inhibited OA progression. *Akkermansia* was identified as the most significantly altered microbiota in response to MICT, and supplementation with *Akkermansia* in destabilization of the medial meniscus mice also alleviated OA. Mechanistically, MICT protects against OA by reducing plasma lactate levels. Production of high levels of lactate by numerous microbiotas was identified as a risk factor induced by destabilization of the medial meniscus. Consistent with these findings, our genetic data revealed that both the knockout of the lactate receptor *Gpr81* and the knockdown of *Gpr81* in articular cartilage with adeno-associated virus 2 mitigated OA progression by increasing anabolic metabolism and suppressing catabolic metabolism. The Gαi-CREB/NF-κB axis was further identified as the primary downstream pathway mediating the biological effects of Gpr81 in chondrocytes. Clinically, we validated a negative correlation between *Akkermansia* abundance and both synovial fluid lactate levels and GPR81 expression in the articular cartilage of OA patients. Collectively, the results of this study not only reveal the mechanism underlying the protective effects of exercise against OA but also identify a novel therapeutic target for OA treatment.

**Figure:**
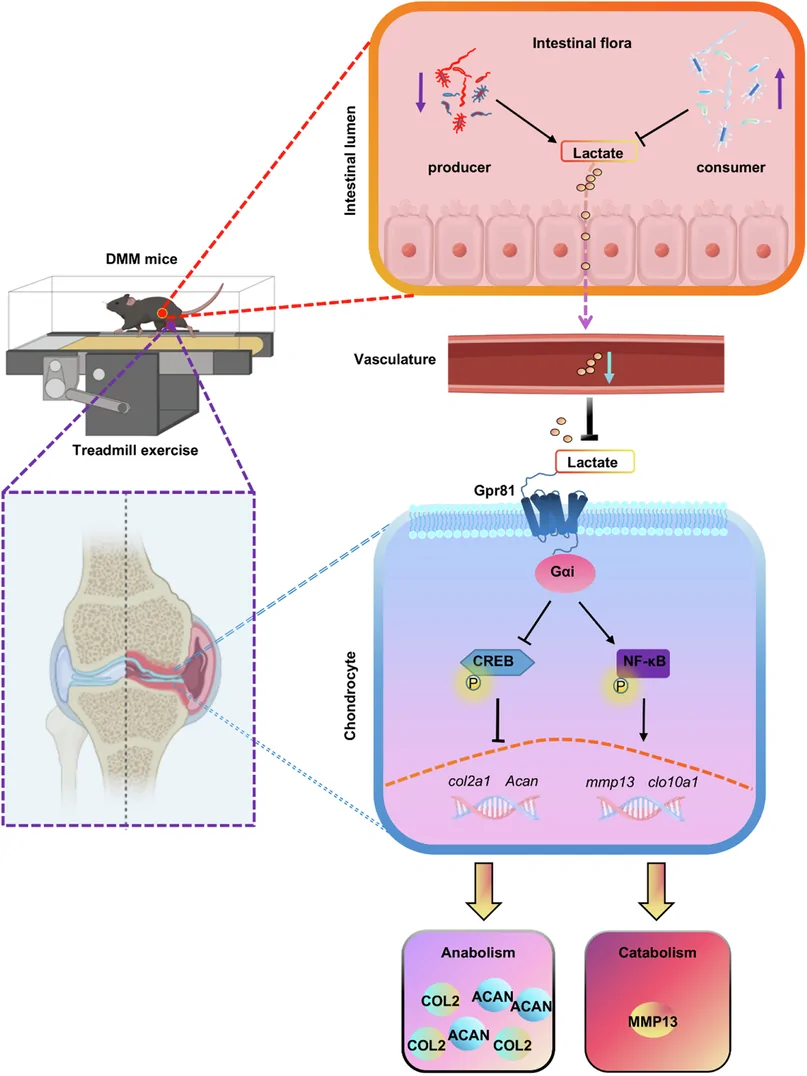
Moderate-intensity continuous training remodelled the gut microbial composition in destabilization of the medial meniscus mice, which in turn decreased blood lactate levels, further alleviated destabilization of the medial meniscus-induced hyper-lactate and Gpr81 overactivation, ultimately ameliorating osteoarthritis by coordinating the Gαi-CREB/NF-κB signalling pathway.

Moderate-intensity continuous training remodelled the gut microbial composition in destabilization of the medial meniscus mice, which in turn decreased blood lactate levels, further alleviated destabilization of the medial meniscus-induced hyper-lactate and Gpr81 overactivation, ultimately ameliorating osteoarthritis by coordinating the Gαi-CREB/NF-κB signalling pathway.

## Exercise Therapy Reduces Osteoarthritis Through Gut Microbiota

Osteoarthritis (OA) is a widespread joint disorder marked by cartilage degeneration, affecting millions globally. This study explores the impact of exercise intensity on OA, focusing on moderate-intensity continuous training (MICT) and its effects on gut microbiota and lactate levels. Researchers used a mouse model to simulate OA and compared the effects of high-intensity continuous training and MICT. MICT significantly alleviated pain, reduced cartilage damage, and improved subchondral bone structure, unlike high-intensity continuous training. The study found that MICT reshapes gut microbiota, reducing systemic lactate levels and suppressing Gpr81-dependent signalling, which is crucial for cartilage homeostasis. These findings suggest that targeting the lactate-Gpr81 axis through MICT could offer a promising strategy for OA treatment. Future research could further explore the role of the gut microbiota in exercise-induced benefits for OA, potentially leading to novel therapeutic approaches.

This summary was initially drafted using artificial intelligence, then revised and fact-checked by the author.

## Introduction

Osteoarthritis (OA), one of the most prevalent joint disorders worldwide, is characterized by progressive cartilage degeneration and synovial hypertrophy^1^. Nearly 528 million people are estimated to be affected by OA globally, making it the 15th leading cause of disability^2^. In addition to inducing chronic joint pain and functional impairment, OA significantly decreases patients’ quality of life^3,4^. Current pharmacological therapies primarily target symptom relief by mitigating pain and slowing cartilage degradation; however, they exhibit limited efficacy in promoting cartilage regeneration^5^. Moreover, the long-term use of these drugs may lead to adverse systemic effects, whereas invasive surgical interventions pose risks such as infection and thrombosis^6,7^. Given these limitations, the development of safer and more effective therapeutic strategies for OA remains an urgent clinical priority.

Exercise therapy has gained recognition as an effective non-pharmacological intervention for various diseases and has demonstrated significant clinical potential. However, the effects of different exercise intensities on OA progression and their underlying mechanisms remain controversial. Previous studies have indicated that high-intensity continuous training (HICT) exacerbates articular cartilage degradation in OA by upregulating matrix metalloproteinase-13 expression and downregulating collagen II expression, whereas moderate-intensity continuous training (MICT) appears to have protective effects^8^. Emerging evidence suggests that MICT may mitigate chondrocyte pyroptosis by promoting autophagy and suppressing inflammation via inhibition of the PI3K/Akt/NF-κB pathway^9,10^; nevertheless, whether MICT influences OA through indirect mechanisms remains unclear. Notably, the gut microbiota plays a pivotal role in bone mechanical adaptation, with studies showing that antibiotic (ABX)-induced microbiota depletion impairs loading-induced bone formation^11^. This raises the intriguing possibility that the gut microbiome may be responsible for the beneficial effects of MICT on OA.

Lactate, a key microbe-derived metabolite, is abundantly secreted by the gut microbiota^12^. Notably, elevated serum lactate levels have been observed in patients with OA compared with healthy controls^13^, indicating that high serum lactate levels may be a contributing factor to OA. Intriguingly, skeletal muscle also generates substantial amounts of lactate during HICT^14^, suggesting that exercise-induced lactate may contribute to OA aggravation. In contrast, MICT has been reported to result in lower circulating lactate concentrations^15^; furthermore, our data showed that MICT could also decrease serum lactate levels in the OA environment, which may explain why it can alleviate OA. As the gut microbiota composition significantly influences systemic lactate levels^16,17^, clarifying how MICT reduces blood lactate levels through gut microbes is crucial for understanding the mechanism of exercise therapy in treating OA.

Gpr81, a well-characterized lactate receptor, was first identified in adipose cells^18^. It has been implicated in various pathological processes, including liver fibrosis^19^ and obesity^20^, and the cAMP‒PKA pathway has been identified as its major downstream signalling cascade^21^. Emerging evidence suggests that Gpr81 plays a regulatory role in macrophage polarization within the bone marrow microenvironment^22,23^. However, the function of Gpr81 in cartilage pathophysiology remains unexplored. Given that lactate — a known ligand of Gpr81 — critically influences cartilage homeostasis, we propose that Gpr81 may serve as a novel therapeutic target for OA. In this study, we hypothesize that MICT attenuates OA progression by reshaping the gut microbiota composition, thereby reducing systemic lactate levels and suppressing Gpr81-dependent signalling. Targeting this lactate-Gpr81 axis may provide a promising strategy for OA intervention.

## Materials and methods

### Animals

The experiments involving mice were reviewed, and approval was granted by the East China Normal University (ECNU) Animal Care and Use Committee (m20201103). *Gpr81* knockout mice (*Gpr81*^*−/−*^) were generated in the C57BL/6 J mouse strain by the Animal Center at East China Normal University using the CRISPR/Cas9 system. In detail, two target sites spaced approximately 100 bp apart were selected on *Gpr81* exon 1 (sgRNA1: TGGAGATATCGCCTGTCGCCTGG; sgRNA2: GGAA CGATTTGCCA TCGATGG). The optimal target site was selected on the basis of off-target rate predictions. A mixture of sgRNA and Cas9 mRNA was microinjected into the cytoplasm of fertilized mouse eggs. After 1–2 h of in vitro culture, viable embryos were transplanted into pseudopregnant female oviducts. Founder mice were genotyped by genomic DNA analysis, and heterozygous offspring were backcrossed with wild type (WT) C57BL/6 J mice to establish the F1 generation. Successful knockout was confirmed by PCR, which revealed a 727 bp deletion in the *Gpr81* gene. Male C57BL/6 J mice, including WT and *Gpr81*-deficient (*Gpr81*^*−/*^^*−*^, C57BL/6 J) mice, were used for the experiments. Genotyping was performed on *Gpr81*^*−*^^*/*^^*−*^ mice using the following forward and reverse primers: forward, 5’-GGGTCGTGCTGTCTCATC G-3’; reverse, 5’-TTGTTGGGCT GTTTGTCTTA-3’.

### Patients with osteoarthritis

Nine patients with primary knee osteoarthritis diagnosed according to the American College of Rheumatology (ACR) clinical and radiological criteria and 15 age-matched (70–80 years old) non-OA controls were recruited from Minhang Hospital in Shanghai, China. Information on the patients with OA is shown in Supplementary Table 1. All human experiments performed in this study were approved by the Ethics Committee of Minhang Hospital of Shanghai (2025-Approval-068-01K). All participants provided written informed consent, and those in the control group were confirmed to be free of joint pain, swelling, dysfunction and radiological OA (Kellgren–Lawrence grade 0). To minimize confounding effects on the gut microbiome, participants were excluded if they met any of the following criteria: history of active gastrointestinal diseases (including inflammatory bowel disease, irritable bowel syndrome, peptic ulcer, acute gastroenteritis or intestinal malignancy) in the past 12 months; ABX use in the past 3 months; use of probiotics, prebiotics or synbiotics in the past 6 months; pregnancy or lactation; severe systemic diseases (e.g. severe cardiac, hepatic or renal failure) or non-OA autoimmune disorders; and history of major gastrointestinal surgery.

### Exercise programme

The mice in the MICT exercise group were subjected to 8 weeks of treadmill running at a constant speed. The protocol included 6 days of exercise per week, 1 day of rest, and unrestricted activity in their cages during non-exercise periods. The adaptive training phase was performed at a speed of 10 m/min for 30 min per day, whereas the formal training phase was conducted at 15 m/min for 60 min per session as previously described^24^. In contrast, the HICT group exercised at a speed of 25 m/min for 60 min per session as previously described^25^. All the animals were euthanized following the completion of the 8-week exercise regimen.

### Faecal microbiota transplantation

Faecal samples from the sedentary and MICT groups were collected and stored at −80 °C during the last two weeks of treadmill exercise. Faecal samples were diluted with anaerobic PBS and filtered through a 0.25-mm stainless steel sieve. The male mice were given ABX in their drinking water for 1 week. The mixture contained ampicillin, metronidazole, neomycin and vancomycin. After a 24-h washout period, each recipient received 200 μL of the donor mixture by gavage three times a week for 8 weeks. Control recipients received sterile PBS.

### Destabilization of the medial meniscus surgery

For the destabilization of the medial meniscus (DMM) model, the medial meniscus on the right knee was destabilized by surgically transecting the medial meniscus ligament according to previously described methods^26,27^; in brief, 8-week-old male C57BL/6 J mice underwent anaesthesia, after which the joint capsule on the right knee lateral to the patellar ligament was removed. The medial meniscotibial ligaments were severed using a microsurgical scalpel. In contrast, the sham-operated mice received incisions at the knee joint without any tissue removal. Pain assessment was performed before the mice were euthanized, and their knee joints were collected for histological analysis.

### Culture and administration of *Akkermansia muciniphila*

*Akkermansia muciniphila* was isolated and incubated following a previously described protocol^28,29^. In brief, fresh faecal samples were obtained from WT mice, and the samples were immediately transferred to anaerobic conditions and diluted in saline containing 0.5 g of L-cysteine-HCl. The faecal solution was subsequently inoculated into mucin medium, and the inoculated medium was incubated at 37 °C under anaerobic conditions for up to 7 days. When bacterial growth was observed, the medium was streaked on mucin agar plates comprising mucin medium plus 1.0% agar. After further incubation at 37 ° C under anaerobic conditions, single colonies were picked and reinoculated into mucin medium. Under strictly anaerobic conditions, the cultured bacteria were washed thoroughly and concentrated in anaerobic PBS supplemented with 25% (vol/vol) glycerol. These bacterial cultures were then frozen and preserved at −80 °C for long-term storage. To determine the bacterial concentration (colony-forming units, CFU/ml), a representative glycerol stock was thawed under anaerobic conditions, and plate counting was performed on mucin media containing 1% agarose (agar noble; Difco). Prior to oral gavage administration, the glycerol stocks were thawed in an anaerobic environment and diluted with anaerobic PBS to a final concentration of 2×10^8^ CFU/100 µl, with the final glycerol concentration adjusted to 2.5% (vol/vol).

### AAV packaging and purification

For in vivo *Gpr81* knockdown, a *Gpr81*-specific shRNA (target sequence: 5’-CGTGTCTGCTAGACTCTATTT-3, corresponding to MISSION shRNA #2, TRCN0000008942)^30^ and a non-targeting control shRNA were cloned and inserted into an AAV2 vector under the U6 promoter, with an EGFP reporter for tracking infection efficiency (Supplementary Fig. 9). Recombinant AAV2 particles were packaged in 293 T cells: briefly, cells were seeded in 10-cm dishes and transfected at 70–90% confluence using PEI MAX with a 1:1:1 mass ratio of AAV2 shuttle plasmid, rep/cap plasmid and helper plasmid. At 72 h post-transfection, the cell culture supernatants were collected, and the cell pellets were lysed by three cycles of freeze-thawing before being combined with the supernatants. Virions were concentrated by PEG/NaCl precipitation, purified via iodixanol density gradient centrifugation (15%, 25%, 40%, 60% layers), and dialysed against 1× PBS overnight. The virus was further concentrated using a 100-kDa ultrafiltration device, filtered through a 0.22-μm membrane, and stored at −80 °C. Viral titres were determined by quantitative real-time (qRT)‒PCR targeting the AAV ITR region. Under isoflurane anaesthesia, 10 μl of AAV2 (1×10¹¹ vg/ml) was injected into the knee joint cavity.

### Pain assay

The mice were kept in a quiet environment and allowed to acclimatize for at least 60 min. The thermal hyperalgesia of the hind paw was estimated with a hot-plate assay (UGO 37370) as reported previously^31^. An infrared radiant heat source was directed onto the hot plate surface of the hind paw, and the withdrawal response was noted as the paw withdrawal latency. The test was performed by individuals who were blinded to the experimental groups, and the assessment was repeated three times for each animal.

### Intra-articular injection

Intra-articular injections were performed under isoflurane anaesthesia at a fixed volume of 5 μl per knee joint once weekly for 4 consecutive weeks. Lactic acid (Sigma‒Aldrich, W261114) was dissolved in sterile 0.9% saline, filter-sterilized (0.22 μm), and prepared to a final concentration of 10 mM (1.12 mg/ml). Pertussis toxin (PTX; Sigma‒Aldrich, P7208) was dissolved in sterile 0.9% saline to a concentration of 2 μg/ml for local inhibition of Gαi signalling^32^. Mice subjected to DMM surgery were randomly divided into four groups: the saline control group; the 10 mM lactate-treated group; the PTX-treated group; and the PTX plus lactate-cotreated group. All the solutions were freshly prepared before each injection.

### Lactate measurement

All tissue and blood samples were collected at 48 h following the final MICT session for blood lactate concentration detection. As shown in previous reports^33^, serum lactate levels return to baseline values at 48 h after the end of exercise, and the contribution of acute muscle-derived lactate is minimal. Blood lactate levels were measured using an L-lactate assay kit (Abcam, ab65331) in strict accordance with the manufacturer’s instructions.

### Cell culture

Primary articular chondrocytes were obtained by digestion of articular cartilage tissue from newborn mice (day 3 postnatally) with 0.2% type 2 collagenase (Gibco, MA, USA) as previously described^34^. Isolated primary chondrocytes were maintained in DMEM/F12 (Gibco) supplemented with 1% penicillin/streptomycin and 10% (v/v) fetal bovine serum (Gibco). Mouse articular chondrocytes were stimulated with interleukin 1β (IL-1β) (Peprotech, 211-11B-1) for experiments.

### Micro-CT analysis of knee joints

After the mice were euthanized, the knee joints were harvested and fixed in 4% paraformaldehyde (PFA) for 48 h, followed by micro-CT scanning, and micro-CT analysis was used to detect subchondral bone in the mouse knee joints. The knee joints were scanned at 60 kV, 166.0 μA and a resolution of 7 μm per pixel using X-ray microtomography (SKYSCAN 1272, Bruker micro-CT). Three-dimensional reconstruction of the knee joints was performed using CTVOL (Bruker micro-CT).

### 16S ribosomal RNA sequencing

After 8 weeks of treadmill exercise, the caecum content samples were collected into 2-ml sterile cryopreservation tubes and stored at −80 °C. After the total DNA of the sample was extracted, primers were designed according to the conserved region, a sequencing connector was added at the end of the primers, PCR amplification was carried out, the products were purified, quantified and homogenized to form a sequencing library, the constructed library was subjected to library quality control, and the library that passed quality control was sequenced on an Illumina NovaSeq 6000 platform. The raw image data files obtained from high-throughput sequencing (such as those from Illumina NovaSeq and other sequencing platforms) were converted into raw sequenced sequences (sequenced reads) by base calling analysis, and the results were stored in FASTQ (abbreviated as fq) file format, which contains the sequence information of the sequenced reads and their corresponding sequencing quality information.

The 16S ribosomal RNA (rRNA) sequencing analysis procedure comprises several key steps. First, the quality of the second-generation or third-generation sequencing data must be assessed and preprocessed. For second-generation data, the following steps are involved: first, filtering using Trimmomatic; second, primer removal using Cutadapt; third, assembly using USEARCH; and finally, chimera removal. In the context of third-generation data, the process involves the acquisition of circular consensus sequences, the identification of barcodes and primers, filtration by length and the elimination of chimeras. Operational taxonomic unit (OTU)/amplicon sequence variant analysis is then conducted, employing USEARCH clustering for OTUs or QIIME2’s DADA2 method for amplicon sequence variants. Following this, species annotation is performed utilizing databases such as Silva through alignment, naive Bayes classification, or a combination of these methods. A diversity analysis is then conducted, encompassing the calculation of indices and the analysis of correlation curves for alpha diversity, the calculation of a distance matrix, a ranking analysis, and the validation and visualization of beta diversity. Afterwards, an analysis of the significance of the intergroup difference is performed, employing tools such as LefSe and Metastats analysis. Additionally, correlation and association analyses are conducted, including variance inflation factor testing, redundancy analysis/canonical correspondence analysis, variation partitioning analysis and correlation networks. Ultimately, a functional prediction analysis is conducted using Picrust2, BugBase and FAPROTAX, among others. The following text is intended to provide a comprehensive overview of the subject matter. These steps are facilitated by the BMKCloud platform.

### Histological analysis

Upon the conclusion of the study, the knee joints were preserved in formaldehyde at a temperature of 4 °C for 48 h. They were then demineralized in 0.5 mol/l ethylenediaminetetraacetic acid (pH 7.4) for a period of 14 days before being embedded in paraffin. Subsequently, 5-μm-thick sections were obtained from the paraffin-embedded blocks and processed with haematoxylin and eosin and safranin O staining, following conventional methods^35^. The assessment of OA in the medial femoral condyle and medial tibial plateau, along with the combined scores for the femur and tibia, was conducted utilizing the OA Research Society International (OARSI) grading scale, which ranges from 0 to 6. The grading for each sample was as follows: 0 indicated intact cartilage; 0.5 indicated a decrease in safranin O staining without structural alterations; 1 indicated minor fibrillations with no cartilage loss; 2 indicated vertical fissures reaching the subsuperficial layer with partial loss of the surface layer; 3 indicated vertical fissures or erosion affecting less than 25% of the articular surface to the calcified cartilage; 4 indicated vertical fissures or erosion affecting 25% to 50% of the articular surface to the calcified cartilage; 5 indicated vertical fissures or erosion affecting 50% to 75% of the articular surface to the calcified cartilage; and 6 indicated vertical fissures or erosion affecting more than 75% of the articular surface to the calcified cartilage. We scored articular cartilage OARSI in osteoarthritic mice according to Glasson et al.^36^ and synovial inflammation scores according to Krenn et al.^37^.

### Immunostaining

The immunohistochemistry procedure was carried out with the assistance of an immunohistochemical kit from Miao Tong Biological, adhering to the guidelines provided by the supplier. To summarize, the paraffin-embedded sections underwent a series of dehydrating steps before being stabilized in 4% PFA for 30 min. The sections were permeabilized with a 0.1% solution of Triton X-100 and PBS for a period of 30 min, followed by antigen retrieval using 20 μg/ml proteinase K for 20 min. The intrinsic peroxidase reaction was inhibited by the application of 3% hydrogen peroxide (H_2_O_2_); then, the tissue samples were treated with a 2% solution of bovine serum albumin for 60 min to block non-specific binding. Afterwards, the sections were left to react with anti-matrix metallopeptidase 13 (MMP13) antibodies, which were then diluted to a ratio of 1:100, at a constant temperature of 4 °C for an extended period of time overnight. For immunofluorescence staining, the sections were incubated overnight at 4 °C with primary antibodies against collagen type X alpha 1 (COL10A1, 1:100; Abcam, ab260040) and aggrecan (ACAN, 1:100; Proteintech, 13880-1-AP). The sections were then washed and incubated with a fluorescent secondary antibody for 1 h in the dark. The cell nuclei were visualized with 1 μg/ml 4,6-diamino-2-phenylindole (Sigma‒Aldrich, D9542). Three-dimensional images were captured using an Olympus fluorescence microscope (Olympus Corporation, Tokyo, Japan) and counted using ImageJ software.

### Western blotting

Cells were disrupted with radioimmunoprecipitation assay buffer supplemented with a protease inhibitor mix and phosphatase inhibitors from Roche on ice for 15 min. Afterwards, insoluble components were removed via centrifugation at 12,000 rpm for 10 min at 4 °C using an Eppendorf centrifuge (model 5418 R, manufactured in China). The supernatant fluid was then harvested. Protein concentrations were quantified using a bicinchoninic acid kit provided by Thermo Fisher Scientific. Equivalent protein quantities were resolved on 10% or 15% gradient sodium dodecyl sulfate‒polyacrylamide gels from Bio-Rad and transferred onto nitrocellulose membranes supplied by Beyotime. These membranes were pretreated with a 5% bovine serum albumin solution (Beyotime), followed by incubation with a panel of specific primary antibodies, namely, anti-GAPDH (CST, USA, 2118, 1:10,000), anti-MMP13 (Servicebio, GB11247-1, 1:1000), anti-COL2A1 (Proteintech, 86139-2-RR, 1:1000), anti-p-CREB (Cell Signaling Technology, 87G3, 1:2000), anti-CREB (Santa Cruz Biotechnology, sc-25785, 1:2000), anti-NF-κB p65 (Cell Signaling Technology, 8242, 1:1000) and anti-NF-κB p-p65 (Cell Signaling Technology, 3033, 1:1000) antibodies, followed by incubation with secondary antibodies conjugated to horseradish peroxidase from LICOR (catalogue numbers 926-32211/926-32210).

### Quantitative real-time‒PCR

Quantitative reverse-transcription PCR (qRT-PCR) was conducted to assess the comparative mRNA expression levels. Total RNA was extracted from chondrocytes utilizing TRIzol reagent (Invitrogen, 15596026CN). The synthesis of complementary DNA was achieved with a Prime Script RT enzyme kit (Yeasen, catalogue number Drr036a), and real-time PCR analysis was performed using a real-time PCR system (QuantStudio Design & Analysis) with qPCR SYBR Green Master Mix (YEASEN, 11202ES03). The primers used for real-time PCR were as follows:

Mouse *Mmp13*: 5′-GTGTGGAGTTATGATGATGT-3′,

Antisense 5′-TGCGATTACTCCAGATACTG-3′;

Mouse *Col2a1*: 5′-CCTCCGTCTACTGTCCACTGA-3′,

Antisense 5′-ATTGGAGCCCTGGATGAGCA-3′.

### Alcian blue staining

The cultured cells were first washed with PBS and then fixed with PFA for 10 min, after which they were incubated with an Alcian blue staining solution (consisting of 1.0% Alcian blue dissolved in 0.1 N hydrochloric acid) at 4 °C overnight. Unbound dye was removed by rinsing with distilled deionized water. The cultures stained with Alcian blue were subsequently eluted using a solution of 6 M guanidine hydrochloride, and the absorbance was quantified at a wavelength of 620 nm.

### Statistical analysis

Data analysis was carried out utilizing Prism 10.0 software from GraphPad Prism. The results are presented as the mean ± standard deviation (SD). In comparisons involving three or more groups, provided that the data met the assumption of a normal distribution, we employed analysis of variance (ANOVA). We used one-way ANOVA followed by Dunnett’s post hoc test for comparisons involving only one independent variable and two-way ANOVA followed by Dunnett’s post hoc test for experimental designs with two independent variables. Unpaired two-tailed Student’s *t* test was used to compare experiments with only two groups. *P* values less than 0.05 were considered to indicate statistical significance.

## Results

### Moderate-intensity continuous training shows better potential for osteoarthritis treatment than high-intensity continuous training

**Fig. 1:**
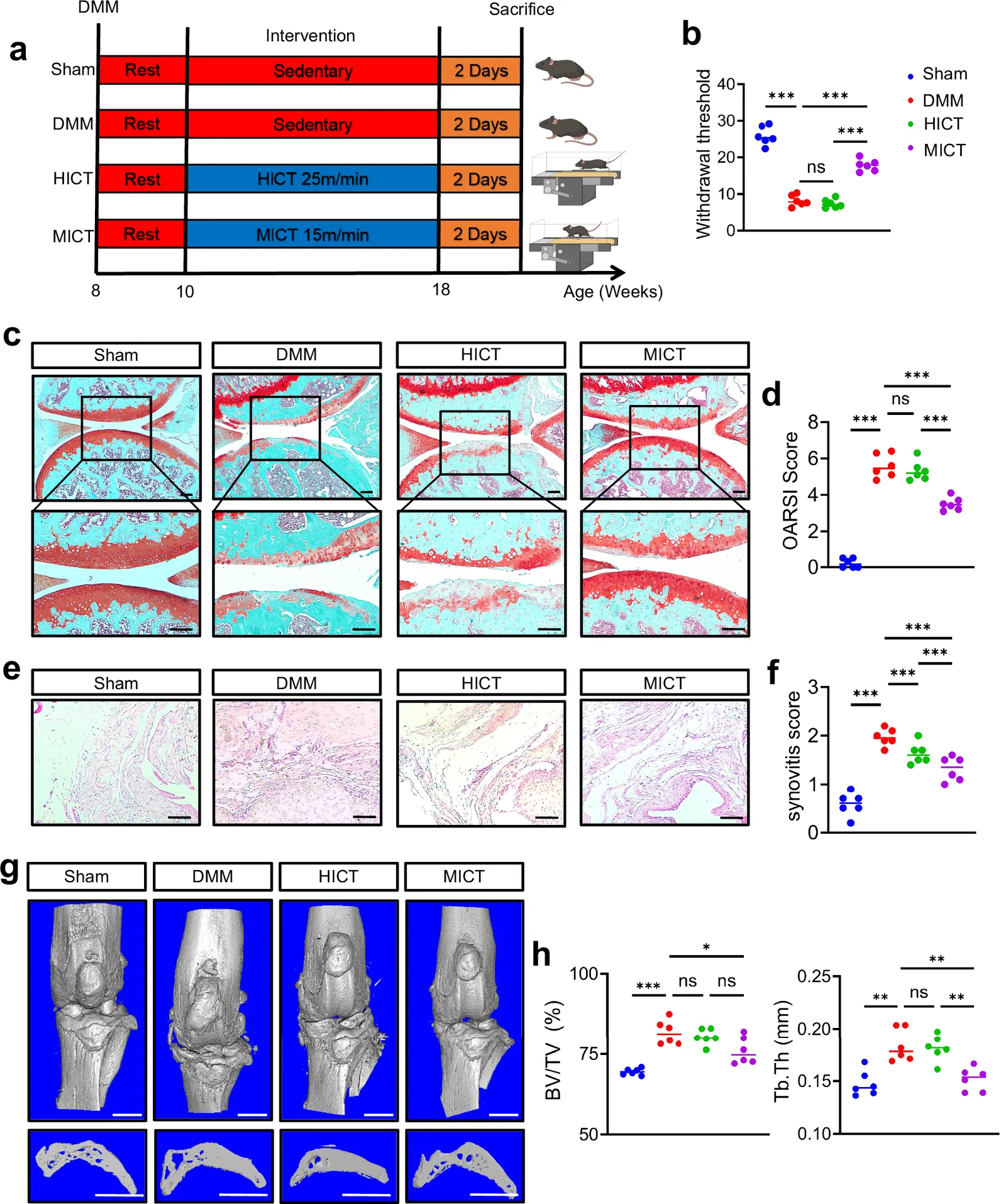
Moderate-intensity continuous training shows better potential for osteoarthritis treatment than high-intensity continuous training. **a**, Graphical overview of experimental design. 24 male mice were randomly divided into four groups. Sham group mice only have the joint cavity opened, followed by sedentary sitting; destabilization of the medial meniscus (DMM) group mice had the medial meniscus freed, followed by sedentary sitting; high-intensity continuous training (HICT) group mice rested for 2 weeks after the DMM surgery, followed by 8 weeks of exercise at a speed of 25 m/min; moderate-intensity continuous training (MICT) group mice rested for 2 weeks after the DMM surgery, followed by 8 weeks of exercise at a speed of 15 m/min of exercise. **b**, Withdrawal threshold after HICT or MICT intervention (*n* = *6*). **c**, Representative images of Safranin O-Fast Green staining of articular cartilage after HICT or MICT intervention, Scale bar = 100 μm. **d**, Osteoarthritis Research Society International (OARSI) scores based on the Safranin O-Fast Green staining histology analysis (*n* = *6*). **e**, Representative images of haematoxylin and eosin staining of articular cartilage after HICT or MICT intervention. Scale bar = 100 μm. **f**, The synovial inflammation score is based on haematoxylin and eosin staining images (*n* = *6*). **g**, Representative 3D reconstructed micro-CT images of knee joint (top) and subchondral bone (bottom) after sports intervention. top: scale bar = 1 mm; bottom: scale bar = 200 μm. **h**, Quantitative analysis of structural parameters of subchondral bone (*n* = *6*). *P < 0.05, ** *P* < 0.01, *** *P* < 0.001；ns, not significant. All data are presented as mean ± standard deviation. Two-way analysis of variance followed by Dunnett’s post hoc test was used for multiple comparisons. BV, bone volume; TV, total volume.

To investigate the differential effects of varying exercise intensities on the pathological improvement of OA, we first performed DMM surgery on 8-week-old male mice to simulate OA progression. After a 2-week recovery period, the mice were assigned to receive 8 weeks of either HICT or MICT (Fig. 1a). We first found that the pain withdrawal threshold was significantly greater in MICT mice than in DMM mice. In contrast, no significant effect on the pain withdrawal threshold was observed in HICT mice (Fig. 1b). Compared with those in DMM mice, cartilage damage and synovial inflammation in MICT-treated mice were significantly alleviated; in contrast, no significant effect on cartilage damage was observed in HICT mice (Fig. 1c–f). Pathological changes in the subchondral bone are closely associated with the progression of OA, and the increase in the bone volume/total volume (BV/TV) ratio of the subchondral bone in DMM mice started at 4 weeks after surgery and was further increased at 6 weeks after surgery^38^. To investigate the effects of different intensities of exercise on the subchondral bone of DMM mice, we analysed the characteristics of the subchondral bone of DMM mice using micro-CT. Our results revealed no significant changes in subchondral bone parameters following 8 weeks of HICT intervention. In contrast, the BV/TV ratio and Tb.Th in DMM mice were significantly reduced according to MICT (Fig. 1g, h; Supplementary Fig. 1), which indicated that MICT could indeed reconstitute subchondral bone and alleviate OA progression in DMM mice.

### Moderate-intensity continuous training prevents osteoarthritis by regulating cartilage metabolic homeostasis driven by the gut microbiota

**Fig. 2:**
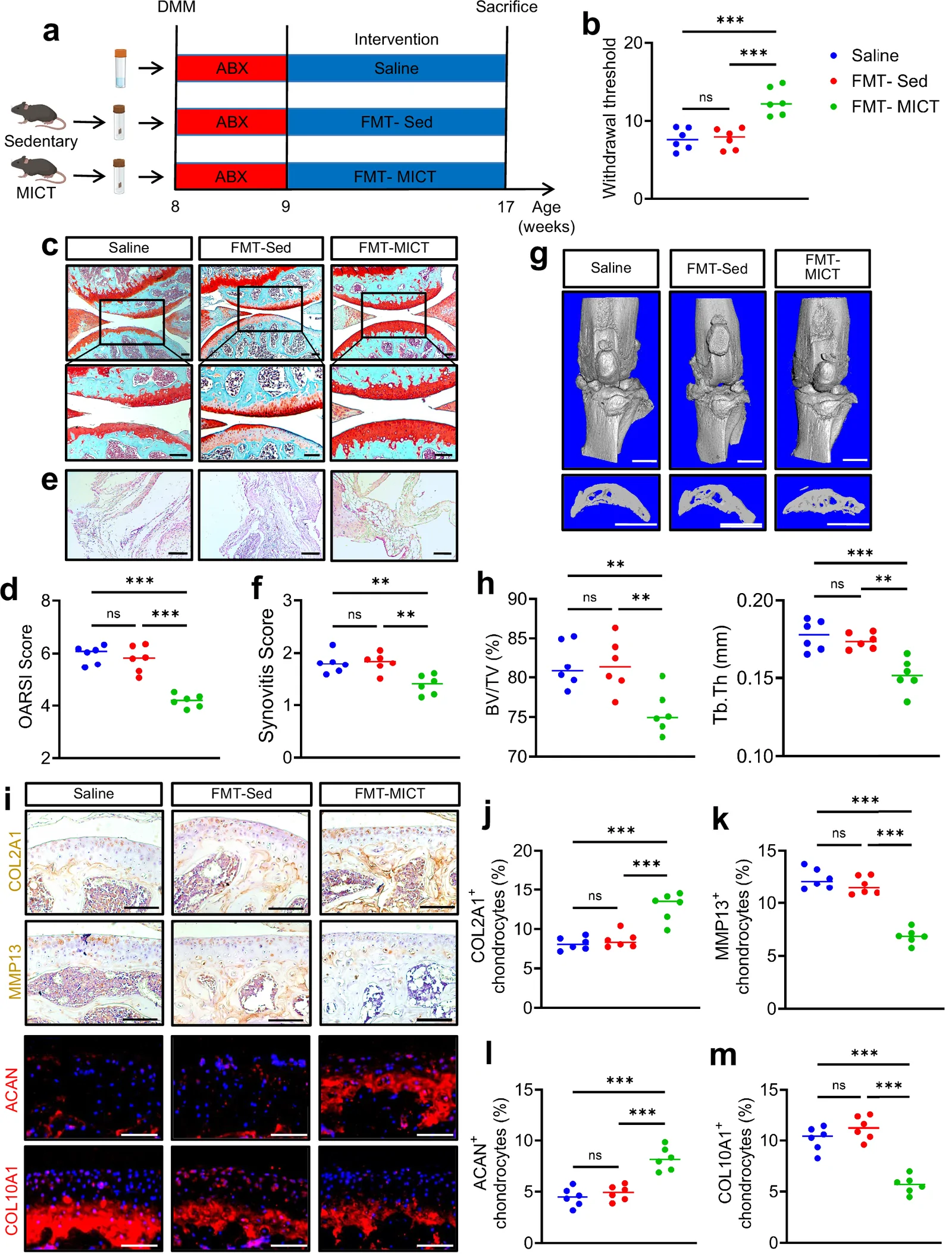
Destabilization of the medial meniscus-induced osteoarthritis progression is alleviated via transplantation gut microbiota from moderate-intensity continuous training mice. **a**, The schematic diagram of the faecal microbiota transplantation (FMT) experiment. All recipient mice were treated with antibiotics for 7 days and then administered saline or transplanted with faecal microbiota from the moderate-intensity continuous training (MICT) mice and sedentary mice for 8 weeks. **b**, Withdrawal threshold in saline, FMT-Sed and FMT-MICT groups (*n* = *6*). **c**, Representative images of Safranin O-Fast Green staining of articular cartilage in the saline, FMT-Sed and FMT-MICT groups, scale bar = 100 μm. **d**, OARSI scores based on the Safranin O-Fast Green staining histology analysis (*n* = *6*). **e**, Representative images of haematoxylin and eosin staining in the saline, FMT-Sed and FMT-MICT groups, scale bar = 100 μm. **f**, The synovial inflammation score is based on haematoxylin and eosin staining images (*n* = *6*). **g**, Representative 3D reconstructed micro-CT images of the knee joint (top) and subchondral bone (bottom) in the saline, FMT-Sed and FMT-MICT groups. Top: scale bar = 1 mm; bottom: scale bar = 200 μm. **h**, Quantitative analysis of structural parameters of subchondral bone (*n* = 6). **i**, Representative immunofluorescence or immunohistochemistry images for COL2A1, MMP13, ACAN and COL10A1 expression in articular cartilage of mice in the saline, FMT-Sed and FMT-MICT groups, scale bar = 100 μm. **j–m**, Percentage of COL2A1^+^ (**j**), MMP13^+^ (**k**), ACAN^+^ (**l**) and COL10A1^+^ (**m**) chondrocytes in articular cartilage of samples shown in (**i**) (*n* = *6*). *P < 0.05, **P < 0.01, ***P < 0.001; ns, not significant. All data are presented as mean ± standard deviation; one-way analysis of variance followed by Dunnett’s post hoc test was used for multiple comparisons. BV, bone volume; DMM, destabilization of the medial meniscus; OARSI, Osteoarthritis Research Society International; TV, total volume.

To further elucidate the mechanism underlying the MICT-mediated regulation of OA progression and given the critical role of the gut microbiota in supporting exercise-induced skeletal health^11^, we hypothesized that MICT protects against OA development by modulating the composition and abundance of the gut microbiota. First, the faecal microbiota from sedentary mice or mice subjected to 8 weeks of MICT was transplanted into DMM mice via oral gavage, whereas control mice received saline via oral gavage (Fig. 2a). The pain withdrawal threshold was significantly greater in faecal microbiota transplantation (FMT)‑MICT‑treated DMM mice than in the saline and FMT‑Sed groups (Fig. 2b). Further histological staining revealed that the OARSI score was markedly lower in the FMT-MICT groups than in the saline and FMT-Sed groups (Fig. 2c, d). The synovitis score of the FMT-MICT group was also lower than those of the saline and FMT-Sed groups, no significant differences were detected between the saline and FMT-Sed groups (Fig. 2e, f). These findings demonstrated that the gut microbiota derived from MICT‑trained mice effectively inhibited OA progression, suggesting that MICT may regulate OA pathogenesis via modulation of the gut microbiota. To further validate this conclusion, we performed micro‑CT analysis to evaluate changes in the subchondral bone structure following FMT‑Sed and FMT‑MICT. The results revealed that bone mass and trabecular thickness in the subchondral bone were significantly reduced in DMM mice receiving FMT‑MICT (Fig. 2g, h), confirming that the gut microbiota from MICT‑treated mice could also mitigate subchondral bone disorder. We next examined cartilage homeostasis in FMT‑treated mice by analysing the expression of the anabolic and catabolic genes of the chondrocyte. FMT‑MICT treatment significantly upregulated the expression of cartilage anabolic markers, including collagen type II alpha-1 chain (COL2A1) and aggrecan (ACAN), whereas it downregulated the expression of the catabolic marker matrix metalloproteinase-13 (MMP13) and the hypertrophic marker collagen type X alpha-1 chain (COL10A1) (Fig. 2i–m). Collectively, these results further support the notion that MICT promotes cartilage anabolism and suppresses catabolism by modulating the gut microbiota, thereby alleviating OA progression.

**Fig. 3:**
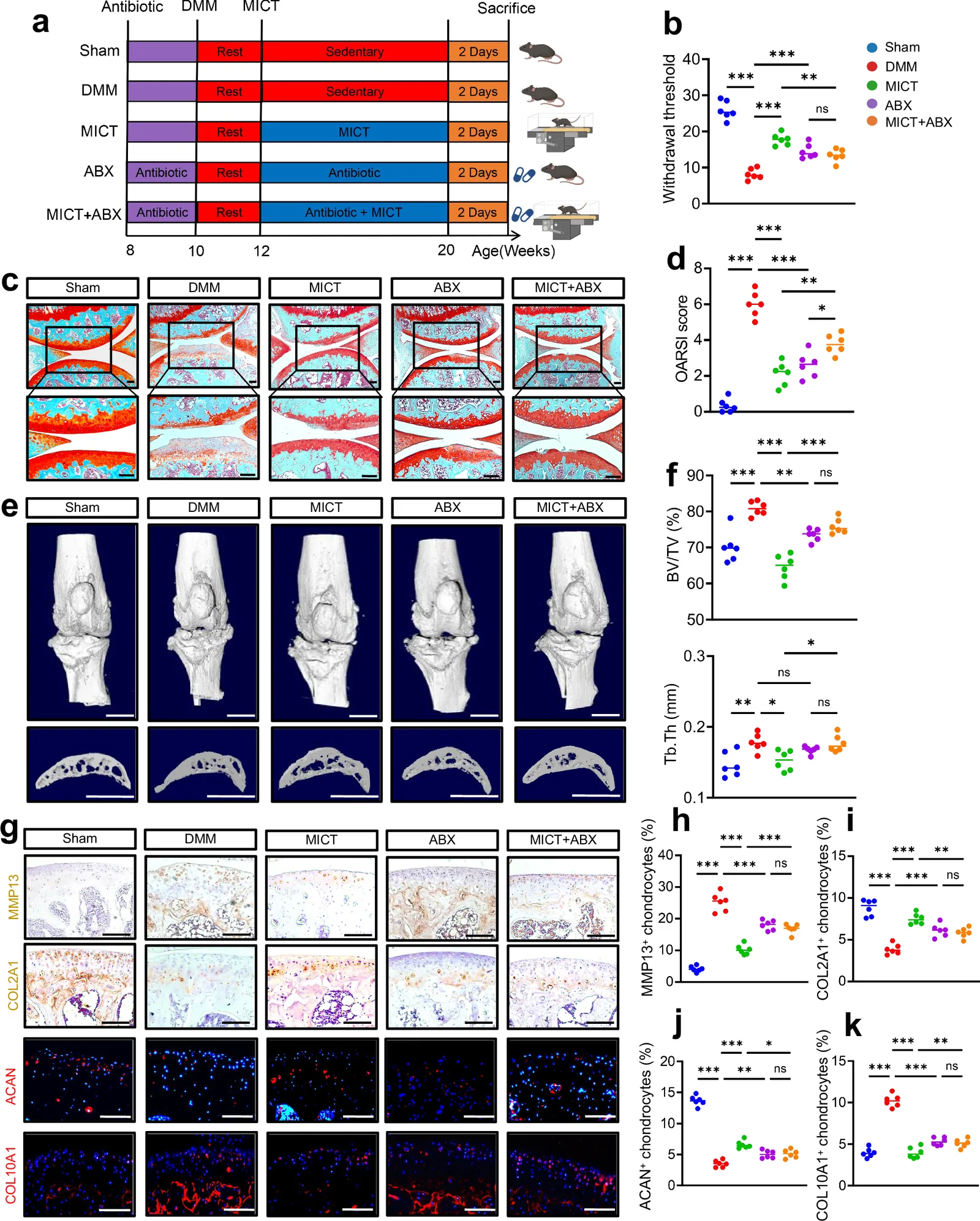
Beneficial effects of moderate-intensity continuous training on destabilization of the medial meniscus-induced osteoarthritis progression were abolished by microbiota-depletion in mice. **a**, Graphical overview of the experimental design. All mice were subjected to destabilization of the medial meniscus (DMM) surgery except those in the sham groups. Mice in the antibiotics (ABX) and moderate-intensity continuous training (MICT) + ABX groups were subjected to DMM surgery after 2 weeks of ABX feeding. Mice in the MICT + ABX and MICT groups were subjected to treadmill exercise at a speed of 15 m/min for 8 weeks. The ABX intervention was continued until the end of the experiments. **b**, Withdrawal threshold in the sham, DMM, MICT, ABX and MICT + ABX groups (*n* = *6*). **c**, Representative images of Safranin O-Fast Green staining of articular cartilage in the sham, DMM, MICT, ABX and MICT + ABX groups, scale bar = 100 μm. **d**, Osteoarthritis Research Society International (OARSI) scores based on the Safranin O-Fast Green staining histology analysis shown in **c** (*n* = *6*). **e**, Representative 3D reconstructed micro-CT images of knee joint (top) and subchondral bone (bottom) in sham, DMM, MICT, ABX and MICT + ABX groups. Top: scale bar = 1 mm; bottom: scale bar = 200 μm. **f**, Quantitative analysis of structural parameters of subchondral bone shown in **e** (*n* = *6*). **g**, Representative immunofluorescence or immunohistochemistry images for COL2A1, MMP13, ACAN and COL10A1 expression in articular cartilage of mice in the sham, DMM, MICT, ABX and MICT + ABX groups, scale bar = 100 μm. **h–k**, Percentage of MMP13^+^ (**h**), COL2A1^+^ (**i**), ACAN^+^ (**j**) and COL10A1^+^ (**k**) chondrocytes in articular cartilage samples shown in **g** (*n* = 6). All data are presented as mean ± standard deviation. *P < 0.05, ** *P* < 0.01, *** *P* < 0.001；ns, not significant. Two-way analysis of variance followed by Dunnett’s post hoc test was used for multiple comparisons. BV, bone volume; TV, total volume.

To further validate the role of the gut microbiota in OA regulation by MICT, we generated pseudo sterile mice by ABX feeding and then performed MICT for 8 weeks with ABX feeding in DMM mice (MICT + ABX) (Fig. 3a). Our results showed that MICT could notably increase the pain withdrawal threshold in DMM mice. Notably, these beneficial effects were abrogated in ABX-treated mice (Fig. 3b). Furthermore, the beneficial effects of MICT on cartilage damage and synovial inflammation were abolished by ABX depletion (Fig. 3c, d, and Supplementary Fig. 2b, c). Consistent with these results, the micro-CT results revealed that ABX treatment abrogated the ameliorative effects of MICT on subchondral bone in DMM mice (Fig. 3e, f, and Supplementary Fig. 2a). These results demonstrated that MICT can inhibit OA progression in DMM mice through effects on the gut microbiota. Next, we examined the effects of MICT and microbiota deficiency on cartilage homeostasis by assessing the expression of cartilage catabolic and anabolic proteins in a DMM-induced OA model. Our results revealed that both MICT and ABX treatment upregulated ACAN and COL2A1 while downregulating MMP13 and COL10A1. Notably, ABX treatment diminished the beneficial effects of MICT (Fig. 3g–k). These results verified our hypothesis that MICT does indeed protect against OA progression by modulating the gut microbiota.

### Moderate-intensity continuous training reduces blood lactate levels in destabilization of the medial meniscus mice by altering the gut microbiota composition

**Fig. 4:**
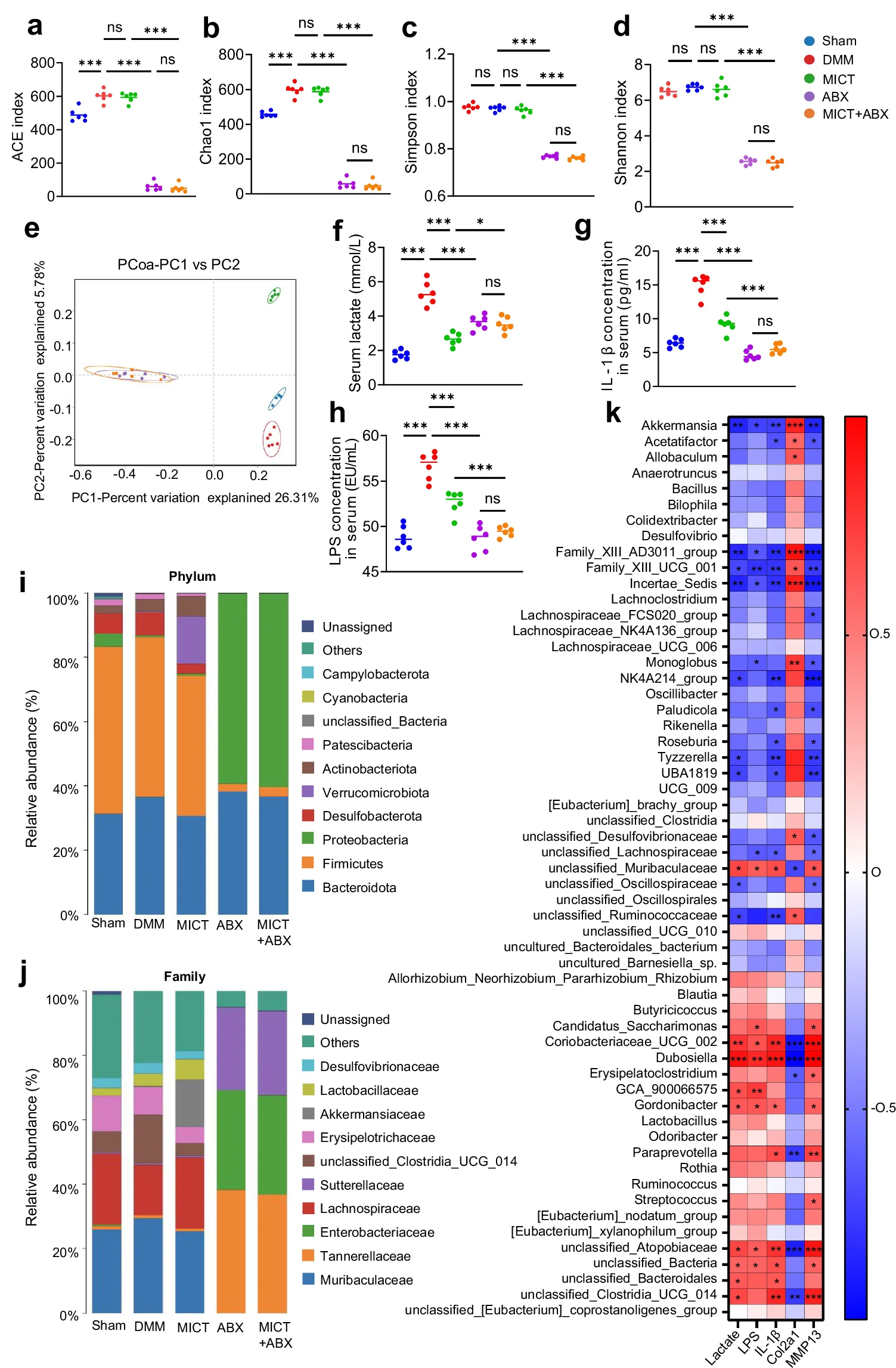
Moderate-intensity continuous training decreases blood lactate level in destabilization of the medial meniscus mice by remodelling lactate metabolism-associated gut microbiota. **a–e**, α- and β-diversity plots to visualize the difference in microbiota structure among the sham, destabilization of the medial meniscus (DMM), moderate-intensity continuous training (MICT), antibiotics (ABX) and MICT + ABX groups. The α-diversity measures with the most common indices (**a–d**) and principal coordinates analysis plots (**e**) showing the β-diversity. **f**, Showing the serum lactate level in the sham, DMM, MICT, ABX and MICT + ABX groups (*n* = 6). **g**, Showing the IL-β concentration level in the sham, DMM, MICT, ABX and MICT + ABX groups (*n* = 6). **h**, Showing the lipopolysaccharide (LPS) concentration level in the sham, DMM, MICT, ABX and MICT + ABX groups (*n* = 6). **i**, Bar chart showing the relative abundance of the top 10 microbiota at the phylum level of caecum content samples. **j**, Bar chart showing the relative abundance of the top 10 microbiotas at the family level of caecum content samples. **k**, DMM-enriched and MICT-responsive microorganisms correlated with serum lactate, LPS and IL-1β as well as articular cartilage COL2A1 and MMP13 levels. All data are presented as mean ± standard deviation. *P < 0.05, ** *P* < 0.01, *** *P* < 0.001；ns, not significant. Two-way analysis of variance followed by Dunnett’s post hoc test was used for multiple comparisons.

We next analysed changes in the abundance and diversity of the gut microbiota across different groups of mice using 16S rRNA sequencing to further explore the underlying mechanisms linking the gut microbiota to OA. A rank abundance curve (RAC) was used to visualize the distribution of species abundance within each community. The results showed that the RACs for the sham, DMM and MICT groups had a wider horizontal axis, indicating a richer species composition within each group. The overall trend towards flattening suggested a more uniform distribution of species across the samples. In contrast, the narrower horizontal axis of the RACs for the ABX and MICT + ABX groups indicated that ABX treatments effectively reduced the abundance of microbial colonies in the mice (Supplementary Fig. 3a). The number of operational taxonomic units (OTUs), as well as all the other measures of α-diversity, was significantly lower in the ABX and MICT + ABX groups than in any of the other groups, confirming the successful modelling of pseudo-sterile mice (Supplementary Fig. 3b). In terms of α-diversity, the ACE and Chao1 indices were significantly greater in DMM mice than in sham-operated mice, whereas no significant difference in the Simpson and Shannon indices was detected between the groups. These results suggested that OA primarily affects microbial community richness rather than community evenness. There were no significant differences between the MICT and DMM groups in any of the α-diversity indices, confirming that MICT intervention does not alter gut microbial diversity (Fig. 4a–d). Principal coordinates analysis of the weighted UniFrac distance matrix and heatmap revealed that samples within the sham, DMM, MICT, ABX and MICT + ABX groups clustered together, indicating that the microbiota composition was similar within each group, and notable differences in the β-diversity of the microbiota in those groups indicated that these factors, including the occurrence of OA, exercise intervention and ABX treatment, can lead to significant differences in the gut microbiota composition between different groups (Fig. 4e).

Kyoto Encyclopedia of Genes and Genomes enrichment analysis revealed enrichment on global and overview maps, indicating differences in overall metabolic flux between the two groups (Supplementary Fig. 3c). To investigate whether the expression of lactate, a key metabolite of glycolysis, is regulated by the gut microbiota, subsequent assays revealed that circulating lactate levels were significantly elevated in DMM mice compared with those in the sham group. Notably, ABX intervention reduced circulating lactate levels in DMM mice, indicating that the gut microbiota modulates systemic lactate homeostasis. To determine whether MICT mitigates DMM-induced lactate elevation in a gut microbiota-dependent manner, we found that gut microbiota depletion partially abrogated the ability of MICT to normalize lactate levels in DMM mice (Fig. 4f). These results indicated that MICT may decrease serum lactate levels by modulating the gut microbiota. IL-1β and lipopolysaccharide (LPS) can also be considered risk factors for OA, whereas the gut microbiota can regulate serum IL-1β and LPS levels^39,40^. To investigate whether the MICT-mediated regulation of IL-1β and LPS is dependent on the gut microbiota, we next analysed the serum levels of IL-1β and LPS in mice cotreated with ABX and MICT. Our results revealed that both MICT and ABX interventions reduced serum IL-1β and LPS levels in DMM mice and that the removal of the gut microbiota not only failed to abolish the ability of MICT to reduce these levels but also exacerbated this decline (Fig. 4g, h).

To further explore whether exercise modulates the effects of gut microbiota composition and abundance on lactate levels and inflammation in DMM mice, we analysed the relative abundance of gut microorganisms across different groups of mice at various taxonomic levels using the biocalcification method (Supplementary Fig. 3d). At the phylum level, the gut microbiota of all groups predominantly consisted of three major phyla: Firmicutes*,* Bacteroidota and Actinobacteria. The abundance of Bacteroidota and Desulfobacterota was effectively reduced, whereas the abundance of Verrucomicrobiota was effectively increased by MICT in DMM mice (Fig. 4i). At the family level, Muribaculaceae*,* Lachnospiraceae and unclassified Clostridia *UCG 014* were the primary targets of MICT in reversing the abnormal microbiota composition in DMM mice, with Akkermansiaceae being the most responsive family to MICT treatment in DMM mice (Fig. 4j). To further identify the key pathogenic gut microbiota associated with OA and the exercise-responsive gut microbiota, we performed clustering analysis of high- and low-abundance species at the genus level, and the results are presented as heatmaps (Supplementary Fig. 4). We correlated the DMM-enriched and MICT-enriched microbiota with inflammation and cartilage metabolism markers. Our analysis revealed that Coriobacteriaceae*_UCG_002, Dubosiella, Erysipelatoclostridium, Paraprevotella,* unclassified_Atopobiaceae*,* and unclassified_Clostridia*_UCG_014* may be key contributors to OA pathogenesis, whereas *Akkermansia*, *Acetatifactor*, Family_XIII*_AD3011*_group, Family_XIII*_UCG_001*, *Incertae_sedis*, *Monoglobus*, and unclassified_Desulfovibrionaceae may represent key probiotics modulated by MICT in OA (Fig. 4k). The abundance of these gut microorganisms correlated significantly with both COL2A1 and MMP13 expression, suggesting their potential roles in inflammation and cartilage metabolism.

### *Akkermansia* can alleviate osteoarthritis in destabilization of the medial meniscus mice by inhibiting cartilage degradation and promoting extracellular matrix generation

**Fig. 5:**
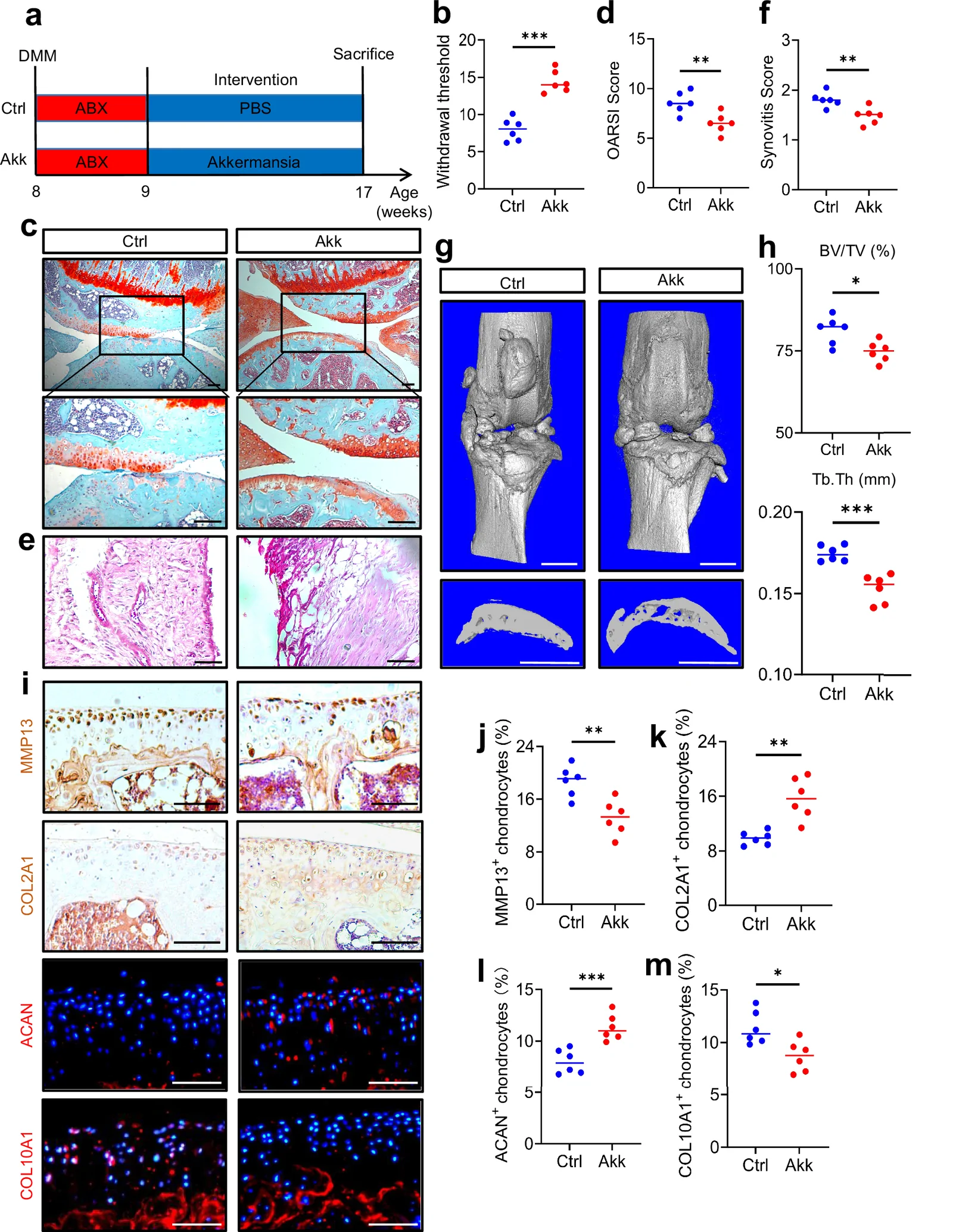
*Akkermansia* improves osteoarthritis in destabilization of the medial meniscus mice by inhibiting cartilage degradation and enhancing extracellular matrix generation. **a**, The schematic diagram of the PBS and *Akkermansia* gavage in destabilization of the medial meniscus (DMM) mice. All recipient mice were treated with antibiotics for 7 days and then administered PBS (control, Ctrl) group or *Akkermansia* (Akk) group for 8 weeks. **b**, Withdrawal threshold in the Ctrl and Akk groups (*n* = *6*). **c**, Representative images of Safranin O-Fast Green staining of articular cartilage in the Ctrl and Akk groups, scale bar = 100 μm. **d**, Osteoarthritis Research Society International (OARSI scores based on the Safranin O-Fast Green staining histology analysis (*n* = *6*). **e**, Representative images of haematoxylin and eosin staining in the Ctrl and Akk groups, scale bar = 100 μm. **f**, The synovial inflammation score based on haematoxylin and eosin staining images (*n* = *6*). **g**, Representative 3D reconstructed micro-CT images of the knee joint (top) and subchondral bone (bottom) in the Ctrl and Akk groups. Top: scale bar = 1 mm; bottom: scale bar = 200 μm. **h**, Quantitative analysis of structural parameters of subchondral bone (*n* = *6*). **i**, Representative immunofluorescence or immunohistochemistry images for MMP13, COL2A1, ACAN and COL10A1 expression in articular cartilage of mice in the Ctrl and Akk groups, scale bar = 100 μm. **j–m** Percentage of MMP13^+^ (**j**), COL2A1^+^ (**k**), ACAN^+^ (**l**) and COL10A1^+^ (**m**) chondrocytes in articular cartilage of mice in the Ctrl and Akk groups (*n* = *6*). *P < 0.05, ** *P* < 0.01, *** *P* < 0.001. All data are presented as mean ± standard deviation. Statistical significance was determined using the unpaired two-tailed Student’s *t* test. BV, bone volume; TV, total volume.

Our l6S rRNA data indicated that *Akkermansia* is a key exercise-responsive genus, indicating that *Akkermansia* may play an independent role in reducing blood lactate levels and ameliorating OA. To investigate whether *Akkermansia* could ameliorate OA, we treated DMM mice with the prepared *Akkermansia* for 8 weeks (Fig. 5a). Our results demonstrated that OA-induced pain (Fig. 5b) and OARSI scores were significantly reduced in the *Akkermansia*-treated (Akk) group (Fig. 5c, d). Synovial inflammation was significantly alleviated in the Akk group (Fig. 5e, f). In addition, micro‑CT analysis revealed that the subchondral bone mass and trabecular thickness significantly decreased in the Akk group (Fig. 5g, h). To explore the role of *Akkermansia* in maintaining cartilage homeostasis, immunostaining assays were performed, which revealed that the administration of *Akkermansia* downregulated the expression of MMP13 and COL10A1 while upregulating the expression of COL2A1 and ACAN (Fig. 5i–m). Collectively, these findings indicate that 8 weeks of intervention with *Akkermansia* can alleviate the progression of OA in DMM mice.

### Lactate/Gpr81 exacerbates overactive bladder in destabilization of the medial meniscus mice by promoting cartilage degradation and inhibiting extracellular matrix generation

**Fig. 6:**
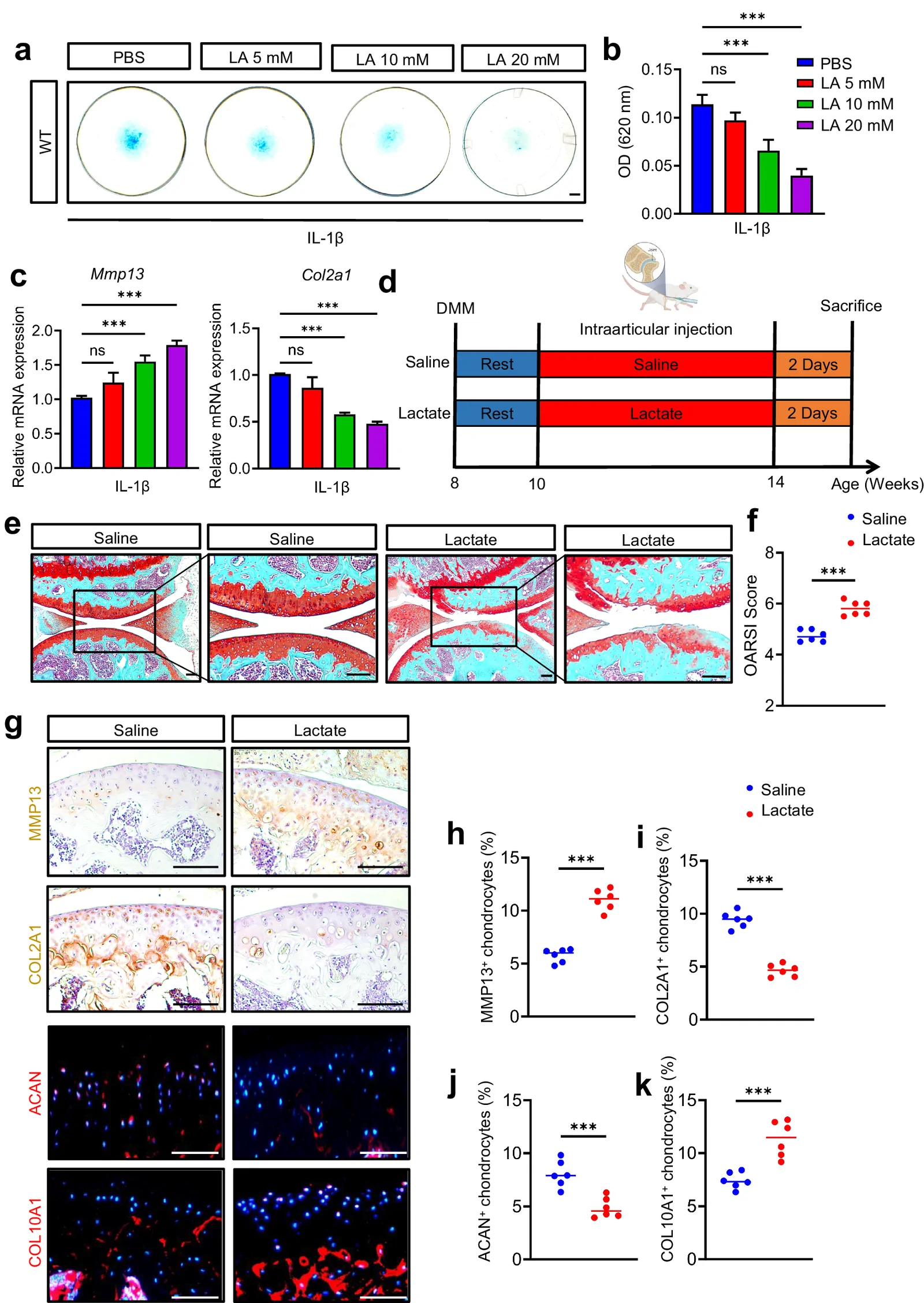
The progression of osteoarthritis in destabilization of the medial meniscus mice was exacerbated by intra-articular injection of lactate. **a**, Bone-marrow mesenchymal stem cells were isolated from wild type mice. The bone-marrow mesenchymal stem cells were cultured in the chondrogenic media with IL-1β (1 ng/ml) for 7 days and stained with Alcian blue, scale bar = 1 mm. **b**, Alcian blue-stained cells were quantified in **a** (*n* = *3*). **c**, Quantitative real-time PCR analysis of catabolic factors *Mmp13* and anabolic factors *col2a1* expressed in chondrocytes treated with PBS, 5 mM lactate (LA), 10 mM LA and 20 mM LA for 7 days (*n* = *3*). **d**, Graphical overview of intra-articular injection. Saline and LA were injected into the articular cavity after DMM surgery. **e**, Representative images of Safranin O-Fast Green staining of articular cartilage after saline and LA were injected into the articular cavity, scale bar = 100 μm. **f**, Osteoarthritis Research Society International (OARSI) scores based on Safranin O-Fast Green staining shown in **e** (*n* = *6*). **g**, Representative immunofluorescence or immunohistochemistry images for MMP13, COL2A1, ACAN and COL10A1 expression in articular cartilage of mice after saline and LA were injected into the articular cavity, scale bar = 100 μm. **h–k**, Percentage of MMP13^+^ (**h**), COL2A1^+^ (**i**), ACAN^+^ (**j**) and COL10A1^+^ (**k**) chondrocytes in articular cartilage of mice in the saline and LA groups (*n* = *6*). *P < 0.05, ** *P* < 0.01, *** *P* < 0.001. All data are presented as mean ± standard deviation. Statistical significance was determined using one-way analysis of variance (**b**, **c**) or unpaired two-tailed Student’s *t* test (**f**, **h–k**).

Previous studies have reported that lactate levels are significantly elevated in OA patients^13,41,42^ and that lactate is also produced by intestinal microbial metabolism^43^. Our results indicated that blood lactate levels were higher in the HICT group than in the MICT group following DMM surgery. Additionally, only MICT was able to reduce blood lactate levels in the DMM group (Supplementary Fig. 5a). Moreover, faecal microbial transplantation from MICT-treated mice effectively reduced blood lactate levels in DMM mice (Supplementary Fig. 5b). To investigate the regulatory effects of lactate on chondrocytes, we first isolated articular chondrocytes or bone-marrow mesenchymal stem cells and stimulated them with IL-1β and lactate, and the results revealed that lactate inhibited chondrocyte differentiation in a dose-dependent manner (Fig. 6a, b) while simultaneously downregulating *Col2a1* expression and upregulating *Mmp13* expression (Fig. 6c). To investigate the effect of lactate on the pathological process of OA in vivo, we injected lactate into the joint cavity of DMM mice (Fig. 6d), and the results revealed that lactate exacerbated cartilage degradation in DMM mice while increasing synovial inflammation levels (Fig. 6e, f; Supplementary Fig. 6a, b). Moreover, immunostaining revealed that lactate decreased COL2A1 and ACAN expression while increasing MMP13 and COL10A1 expression in the DMM mouse model (Fig. 6g–k). These results revealed that lactate exacerbates OA by promoting cartilage degradation and inhibiting extracellular matrix synthesis.

Gpr81 was identified as the endogenous receptor of lactate^44^. To investigate whether lactate can regulate the expression of Gpr81, we first found that lactate injection significantly increased Gpr81 expression in cartilage (Supplementary Fig. 6c, d). Moreover, we found that the expression of Gpr81 in articular cartilage increased after HICT and decreased after MICT intervention (Supplementary Fig. 6e, f). These results suggest that lactate can also regulate Gpr81 expression, thereby amplifying its impact on chondrocyte functions via a cascade effect.

### Global *Gpr81* knockout alleviates osteoarthritis in destabilization of the medial meniscus mice by inhibiting cartilage degradation and promoting extracellular matrix generation

**Fig. 7:**
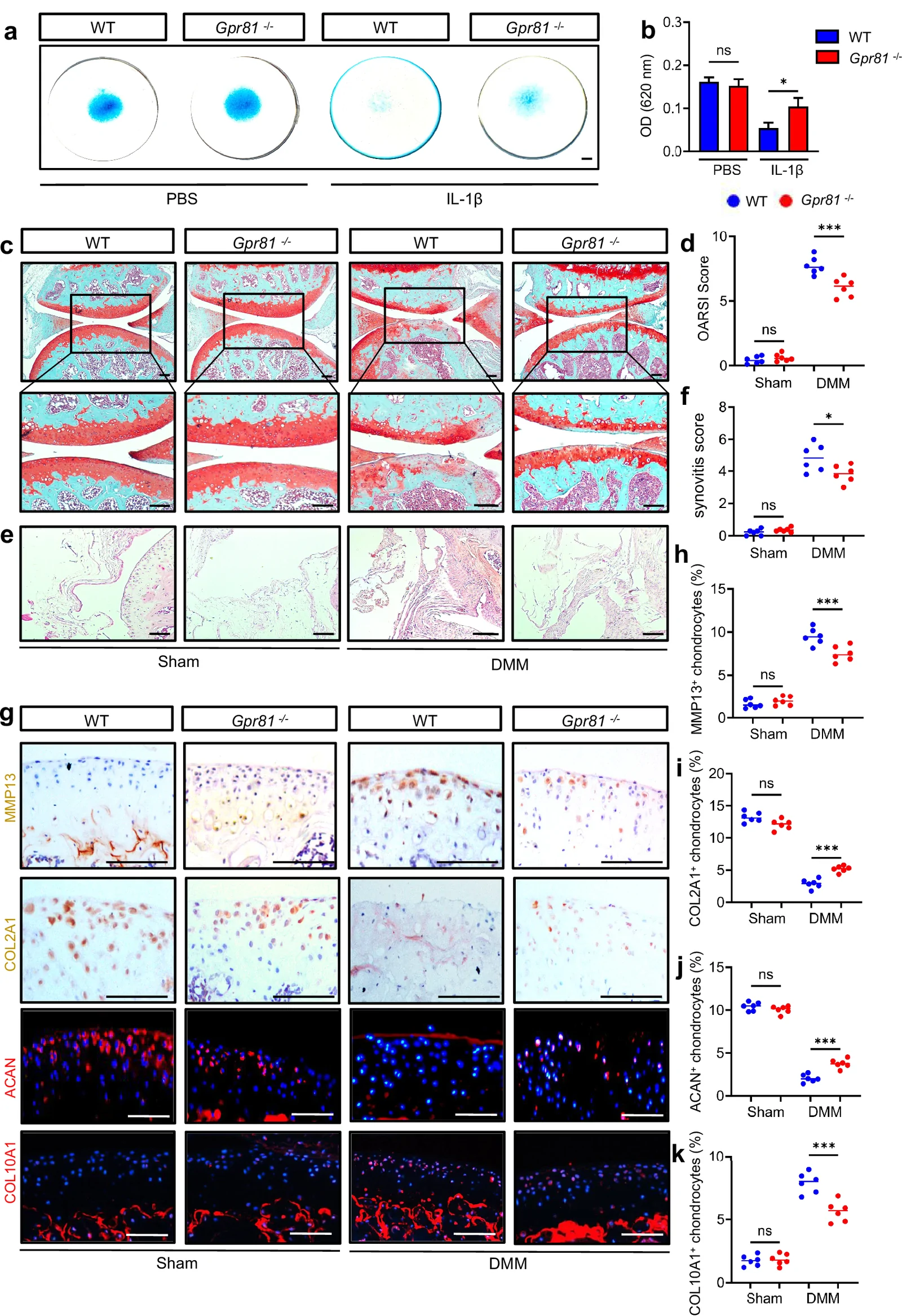
Ablation of *Gpr81* alleviates osteoarthritis progression in destabilization of the medial meniscus mice. **a**, Bone-marrow mesenchymal stem cells were isolated from wild type (WT) and *Gpr81*-deficient mice. The bone-marrow mesenchymal stem cells were cultured in the chondrogenic media with IL-1β (1 ng/ml) for 7 days and stained with Alcian blue, scale bar = 1 mm. **b**, Alcian blue-stained cells were quantified in **a** (*n* = *3*). **c**, Representative images of Safranin O-Fast Green staining of articular cartilage following DMM surgery or sham control operation in *Gpr81*^*−/−*^ and WT mice, scale bar = 100 μm. **d**, Osteoarthritis Research Society International (OARSI) scores based on Safranin O-Fast Green staining (*n* = *6*). **e**, Representative images of haematoxylin and eosin staining on articular cartilage of mice following destabilization of the medial meniscus (DMM) surgery or sham control operation in *Gpr81*^*−/−*^ and WT groups, scale bar = 100 μm. **f**, The synovial inflammation score based on haematoxylin and eosin staining (*n* = *6*). **g**, Representative immunofluorescence or immunohistochemistry images for MMP13, COL2A1, ACAN and COL10A1 expression following DMM surgery or sham control operation in *Gpr81*^*−/−*^ and WT mice, scale bar = 100 μm. **h–k**, Percentage of MMP13^+^ (**h**), COL2A1^+^ (**i**), ACAN^+^ (**j**) and COL10A1^+^ (**k**) chondrocytes in articular cartilage samples shown in **g** (*n* = *6*). *P < 0.05, ** *P* < 0.01, *** *P* < 0.001. All data are presented as mean ± standard deviation. Statistical significance was determined using two-way analysis of variance followed by Dunnett’s test.

We next investigated the relationship between Gpr81 and OA progression by generating *Gpr81*-deficient (*Gpr81*^*−*^^*/−*^) mice. Our findings demonstrated that *Gpr81* knockout promoted chondrogenic differentiation while upregulating *Col2a1* expression and downregulating *Mmp13* expression in chondrocytes under IL-1β stimulation (Fig. 7a, b and Supplementary Fig. 7). Concurrently, the lactate-induced inhibition of chondrocyte differentiation, as well as the upregulation of *Mmp13* and downregulation of *Col2a1*, was abrogated upon *Gpr81* knockout (Supplementary Fig. 8a–c). These findings indicated that lactate does indeed modulate chondrocyte differentiation via Gpr81. Moreover, we also reported that cartilage damage was alleviated in *Gpr81*^*−*^^*/−*^ mice compared with that in their WT controls among DMM mice (Fig. 7c, d). Moreover, the degree of synovitis was decreased in *Gpr81* mice compared with that in their wild-type littermates (Fig. 7e, f). Our data also revealed that the expression of the chondrocyte hypertrophy marker COL10A1 and the cartilage degradation marker MMP13 was lower in *Gpr81*^*-/-*^ mice than in their wild-type littermates. In contrast, the expression of ACAN and COL2A1, which are anabolic cartilage markers, was significantly greater in the articular cartilage of *Gpr81*^*−*^^*/−*^ mice than in that of their WT littermates (Fig. 7g–k). These results demonstrated that the inhibition of Gpr81 may be a new method of OA treatment by decreasing cartilage catabolism and increasing cartilage anabolism.

### Cartilage-targeted *Gpr81* knockdown alleviates osteoarthritis progression in destabilization of the medial meniscus mice by modulating chondrocyte homeostasis

**Fig. 8:**
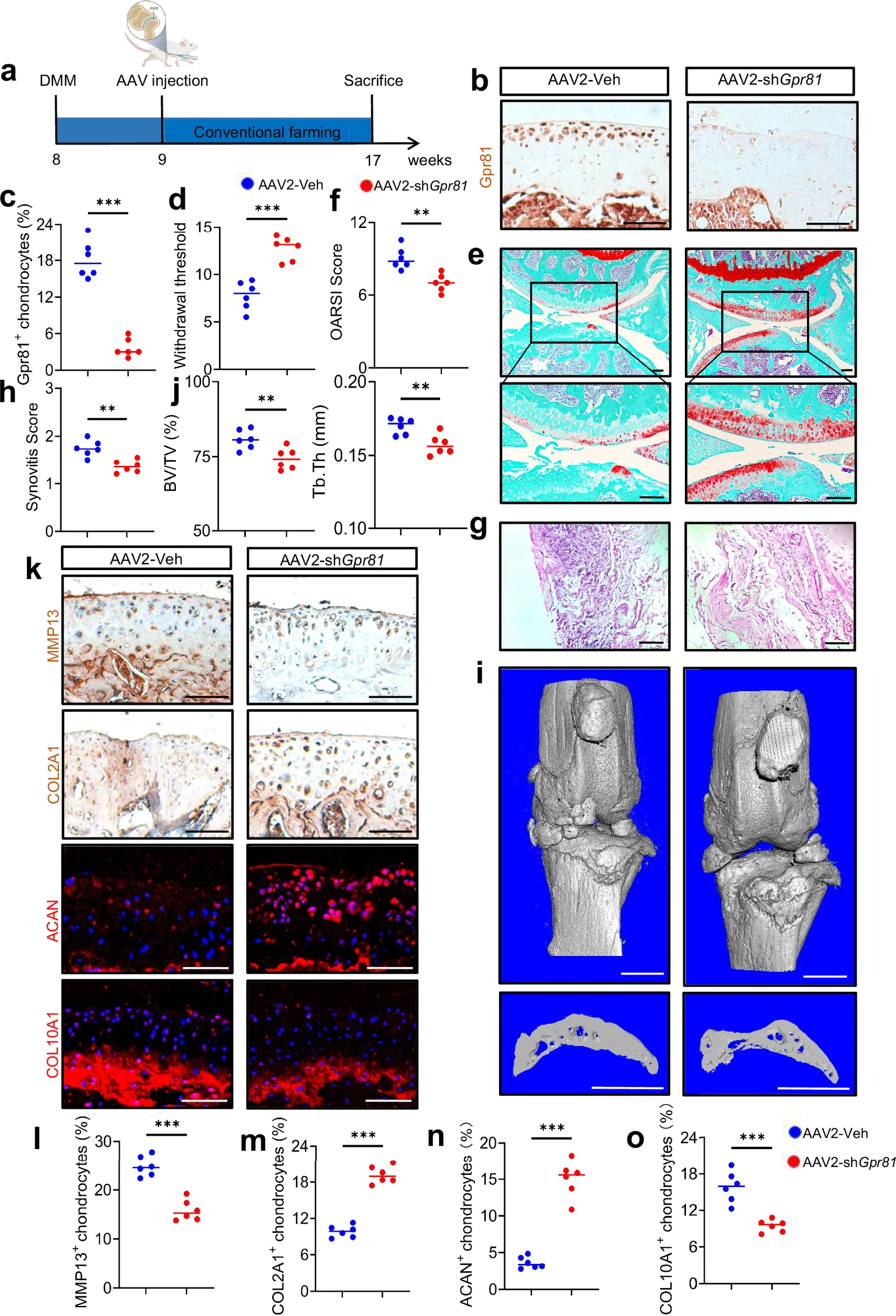
Cartilage-targeted *Gpr81* knockdown alleviates osteoarthritis progression in destabilization of the medial meniscus mice by modulating chondrocyte homeostasis. **a**, Schematic illustration of intra-articular injection of AAV2-Veh and AAV2-sh*Gpr81* in destabilization of the medial meniscus (DMM) mice. 8-week-old mice underwent DMM surgery, followed by a 1-week rest period, after which they received intra-articular injections of AAV2-Veh and AAV2-sh*Gpr81*. **b**, Representative immunohistochemistry images for Gpr81 expression in articular cartilage of mice after intra-articular injections of AAV2-Veh and AAV2-sh*Gpr81*, scale bar = 100 μm. **c**, Percentage of *Gpr81*^*+*^ chondrocytes in articular cartilage samples shown in **b** (*n* = *6*). **d**, Withdrawal threshold in articular cartilage of mice after intra-articular injections of AAV2-Veh and AAV2-sh*Gpr81* (*n* = *6*). **e**, Representative images of Safranin O-Fast Green staining of articular cartilage following DMM surgery or sham control operation in AAV2-Veh and AAV2-sh*Gpr81* mice, scale bar = 100 μm. **f**, Osteoarthritis Research Society International scores based on Safranin O-Fast Green staining (*n* = *6*). **g**, Representative images of haematoxylin and eosin staining on articular cartilage of mice following DMM surgery or sham control operation in the AAV2-Veh and AAV2-sh*Gpr81* groups, scale bar = 100 μm. **h**, The synovial inflammation score based on haematoxylin and eosin staining (*n* = *6*). **i**, Representative 3D reconstructed micro-CT images of the knee joint (top) and subchondral bone (bottom) in the AAV2-Veh and AAV2-*shGpr81* groups. Top: scale bar = 1 mm; bottom: scale bar = 200 μm. **j**, Quantitative analysis of structural parameters of subchondral bone shown in **i** (*n* = *6*). **k** Representative immunofluorescence or immunohistochemistry images for MMP13, COL2A1, ACAN and COL10A1 expression in articular cartilage of mice after intra-articular injections of AAV2-Veh and AAV2-sh*Gpr81*, scale bar = 100 μm. **l–o**, Percentage of MMP13^+^ (**l**), COL2A1^+^ (**m**), ACAN^+^ (**n**) and COL10A1^+^ (**o**) chondrocytes in articular cartilage of mice after intra-articular injections of AAV2-Veh and AAV2-sh*Gpr81* (*n* = *6*). *P < 0.05, ** *P* < 0.01, *** *P* < 0.001. All data are presented as mean ± standard deviation. Statistical significance was determined using unpaired two-tailed Student’s *t* test.

We further investigated the function of *Gpr81* in chondrocyte-mediated OA pathogenesis via intra-articular injection of AAV2 to achieve cartilage-targeted *Gpr81* knockdown (Fig. 8a). AAV2 has been validated to specifically infect cartilage^45^. Our results confirmed that AAV2 efficiently infected articular cartilage at 14 days post-articular injection (Supplementary Fig. 9). We next evaluated the knockdown efficiency of *Gpr81* in cartilage, and the results revealed that AAV2-sh*Gpr81* significantly reduced Gpr81 expression in cartilage, indicating that our animal model was successfully established (Fig. 8b, c). Moreover, compared with control treatment, cartilage-targeted *Gpr81* knockdown via AAV2-sh*Gpr81* markedly alleviated DMM-induced pain (Fig. 8d) and cartilage damage (Fig. 8e, f). Synovial inflammation was also significantly ameliorated following *Gpr81* knockdown (Fig. 8g, h). Micro-CT analysis further revealed that subchondral bone mass and trabecular thickness significantly decreased in AAV2*-*sh*Gpr81* mice (Fig. 8i, j). In addition, knockdown of *Gpr81* via AAV2-sh*Gpr81* significantly decreased the expression of the cartilage catabolic marker MMP13 and the chondrocyte hypertrophic marker COL10A1, but increased the expression of the anabolic markers ACAN and COL2A1 (Fig. 8k–o). These findings demonstrate that cartilage-targeted *Gpr81* knockdown alleviates OA by regulating chondrocyte homeostasis. Collectively, our data provide evidence supporting the involvement of Gpr81 in chondrocyte function during OA pathogenesis.

### Gpr81 regulates cartilage metabolic homeostasis via the Gαi-CREB and NF-κB signalling pathways

**Fig. 9:**
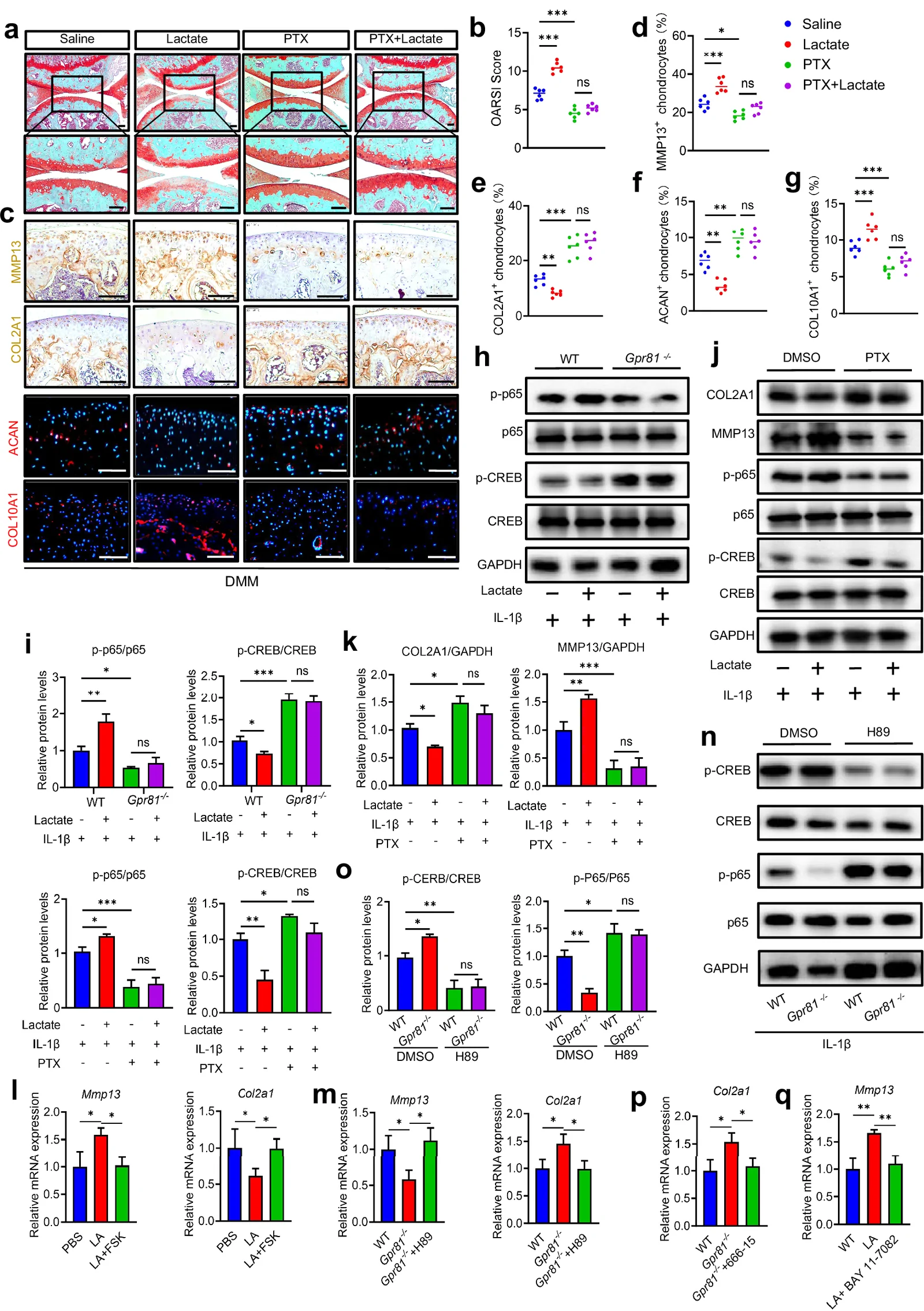
Gpr81 regulates osteoarthritis by activating the Gαi-CREB/NF-κB pathway*.* **a**, Representative image of Safranin O-Fast Green staining of articular cartilage of mice following destabilization of the medial meniscus (DMM) surgery or sham control operation in the lactate (LA), pertussis toxin (PTX) and PTX + LA groups, scale bar = 100 μm. **b**, Osteoarthritis Research Society International (OARSI) scores based on the Safranin O-Fast Green staining (*n* = *6*). **c**, Representative immunofluorescence or immunohistochemistry images for MMP13, COL2A1, ACAN and COL10A1 following DMM surgery in the LA, PTX and PTX + LA groups, scale bar = 100 μm. **d–g**, Percentage of MMP13^+^ (**d**), COL2A1^+^ (**e**), ACAN^+^ (**f**) and COL10A1^+^ (**g**) chondrocytes in articular cartilage samples shown in **c** (*n* = *6*). **h**, Primary articular chondrocytes were isolated from *Gpr81*^*−/−*^ and wild type (WT) knee joints of new born pups, treated with or without LA 30 min under IL-1β (1 ng/ml) stimulation. And p-p65, p65, p-CREB, CREB and GAPDH protein levels were detected by Western blotting. **i**, The quantified results of Western blots shown in **h** (*n* = 3). **j**, Primary articular chondrocytes were isolated from knee joints of newborn pups, treated with or without PTX or LA 30 min under IL-1β (1 ng/ml) stimulation, and COL2A1, MMP13, p-p65, p65, p-CREB, CREB and GAPDH protein levels were detected by Western blotting. **k**, The quantified results of Western blots are shown in **j** (*n* = 3). **l**, Primary articular chondrocytes were isolated from knee joints of newborn pups, treated with or without forskolin (FSK) or LA 30 min under IL-1β (1 ng/ml) stimulation, and *Col2a1* and *Mmp13* mRNA levels were detected using quantitative real-time (qRT)-PCR (*n* = 4). **m**, Primary articular chondrocytes were isolated from WT and *Gpr81*^*−/−*^ knee joints of newborn pups, treated with or without H89 for 30 min under IL-1β (1 ng/ml) stimulation, and *Col2a1* and *Mmp13* mRNA levels were detected using qRT-PCR (*n* = 4). **n**, Primary articular chondrocytes were isolated from knee joints of WT and *Gpr81*^*−*^^*/*^^*−*^ neonatal mice, stimulated with IL-1β (1 ng/ml) and treated with or without H89 for 30 min, and p-p65, p65, p-CREB, CREB and GAPDH protein levels were detected by Western blotting. **o**, The quantified results of Western blots shown in **n** (*n* = 3). **p**, Primary articular chondrocytes were isolated from knee joints of WT and *Gpr81*^*−/−*^ neonatal mice, stimulated with IL-1β (1 ng/ml) and treated with or without 666-15 (a CREB inhibitor) for 30 min. The mRNA level of *Col2a1* was determined using qRT-PCR (*n* = 4). **q**, Primary articular chondrocytes were isolated from knee joints of neonatal mice, stimulated with IL-1β (1 ng/ml) and treated with or without BAY 11-7082 (an NF-κB inhibitor) or LA for 30 min. The mRNA levels of *Mmp13* were determined using qRT-PCR (*n* = 4). *P < 0.05, ** *P* < 0.01, *** *P* < 0.001. All data are presented as mean ± standard deviation. Statistical significance was determined using two-way analysis of variance followed by Dunnett’s test.

Previous studies have shown that Gpr81 is a Gαi-coupled GPCR, indicating that it primarily interacts with the Gαi family of G proteins^46^. To explore whether Gpr81 regulates OA via Gαi signalling, we first performed intra-articular injections of pertussis toxin (PTX), a Gαi inhibitor, in lactate-treated DMM mice. Our results showed that PTX-mediated Gαi inhibition abrogated the lactate-induced exacerbation of OA (Fig. 9a, b). Furthermore, PTX treatment blocked the reduction in cartilage anabolism and the increase in cartilage catabolism driven by lactate in the DMM mice (Fig. 9c–g). These findings demonstrate that Gpr81 regulates OA progression through coupling with Gαi. To further investigate how Gpr81–Gαi modulates cartilage catabolism and anabolism, we reviewed several reports on the regulation of cartilage catabolic and anabolic mechanisms. We found that NF-κB signalling is recognized as a key intracellular pathway involved in cartilage catabolism in chondrocytes^47^. Therefore, we investigated the relationships between lactate, Gpr81, Gαi and NF-κB signalling by stimulating chondrocytes with lactate and PTX. Our data showed that lactate increased the phosphorylation of P65. Knockout of *Gpr81* blocked the lactate-induced increase in P65 phosphorylation (Fig. 9h, i). We also found that inhibition of Gαi by PTX blocked both lactate-induced P65 phosphorylation (Fig. 9j, k). These results suggest that lactate promotes NF-κB activation and cartilage catabolism through the Gpr81–Gαi signalling pathway.

To validate the importance of the lactate–Gpr81–Gαi–cAMP–PKA pathway in regulating chondrocyte metabolism, we first used forskolin to increase cAMP levels in lactate-treated chondrocytes, thereby reversing the lactate-induced increase in Gαi activity and restoring chondrocyte metabolism. These results showed that forskolin effectively alleviated the lactate-induced reduction in chondrocyte anabolism and increase in catabolism (Fig. 9l). More importantly, we then used H89 to inhibit PKA activity in *Gpr81*-deficient chondrocytes, aiming to determine whether PKA, as a key downstream effector of cAMP, mediates *Gpr81*-regulated chondrocyte metabolism. These results demonstrated that H89 effectively abolished the increase in chondrocyte anabolic activity and the suppression of catabolic activity induced by *Gpr81* knockout (Fig. 9m). These two experiments confirmed the critical role of the cAMP‒PKA axis as a key downstream signalling pathway of lactate/Gpr81. To validate the key function of CREB and NF-κB as downstream cAMP–PKA in chondrocytes, we inhibited both the increase in CREB phosphorylation and the decrease in NF-κB phosphorylation induced by *Gpr81* deficiency when PKA was inhibited by H89 (Fig. 9n, o). Subsequently, we treated *Gpr81*-knockout chondrocytes with 666-15, a CREB activation inhibitor, and found that 666-15 blocked the promotion of chondrocyte anabolism driven by *Gpr81* knockout (Fig. 9p). In parallel, to determine whether the NF-κB pathway acts as a key downstream mediator of lactate/Gpr81-driven chondrocyte catabolism, we treated lactate-exposed chondrocytes with the NF-κB inhibitor 11-7028. Our data demonstrated that inhibition of NF-κB signalling abolished the lactate-induced increase in catabolism in chondrocytes (Fig. 9q). These results indicated that CREB and NF-κB are the key modulating factors of lactate/Gpr81–cAMP–PKA signalling downstream. Taken together, our results demonstrated that lactate promotes cartilage catabolism via the Gpr81–Gαi–NF-κB signalling pathway but negatively regulates cartilage anabolism via the Gpr81–Gαi–CREB pathway.

### Clinical data validation of the regulatory role of the gut microbiota–lactate–GPR81 signalling axis in osteoarthritis

**Fig. 10:**
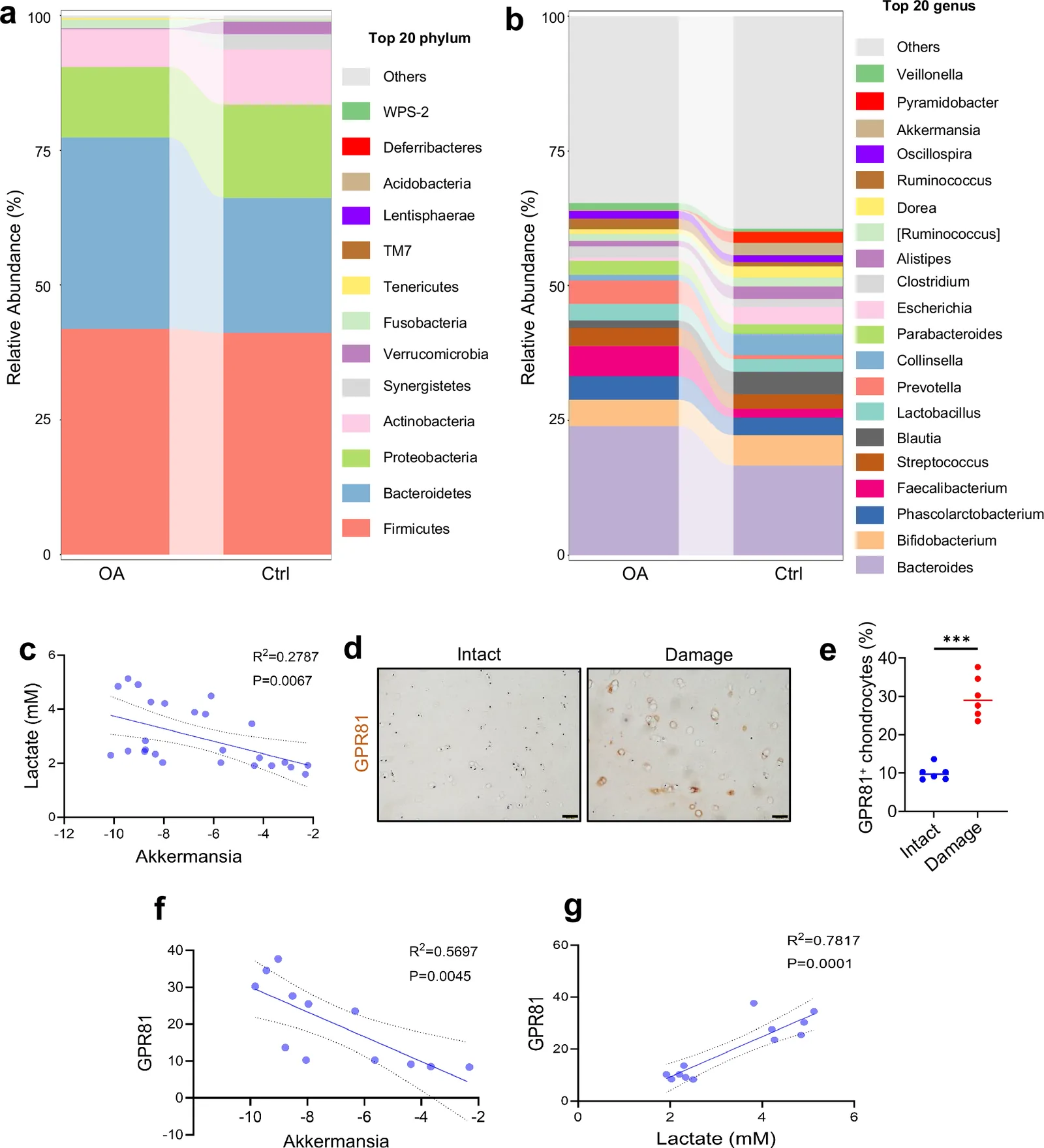
Clinical data validation of the regulatory gut microbiota–lactate–GPR81 signalling axis in osteoarthritis. **a**, Bar chart showing the relative abundance of the top 20 microbiota at the phylum level in faecal samples from patients with osteoarthritis (OA). **b**, Bar chart showing the relative abundance of the top 20 microbiota at the genus level in faecal samples from patients with OA. **c**, Correlation analysis between synovial fluid lactate levels and the relative abundance of *Akkermansia* in patients with OA. R^2^ = 0.2787, *P* = 0.0067. **d**, Representative immunohistochemistry images for GPR81 expression in damaged and intact chondrocytes from clinical samples. Scale bar = 100 μm. **e**, Percentage of GPR81^+^ chondrocytes in articular cartilage samples shown in **d** (*n* = *6*). All data are presented as mean ± standard deviation. ***P < 0.001. Statistical significance was determined using an unpaired two-tailed Student’s *t* test. **f**, Correlation analysis between the percentage of GPR81^+^ chondrocytes in articular cartilage samples and the relative abundance of *Akkermansia* in patients with OA. R^2^ = 0.5697, *P* = 0.0045. **g**, Correlation analysis between the percentage of GPR81^+^ chondrocytes in articular cartilage samples and synovial fluid lactate in patients with OA. R^2^ = 0.7817, *P* = 0.0001. Correlation analysis was performed using Pearsonʼs correlation test. Ctrl, control.

To assess the contribution of the gut microbiota–lactate–GPR81 axis to OA in clinical patients, we collaborated with Minhang Hospital in Shanghai to collect knee cartilage synovial fluid and faecal samples from patients with OA, as well as from age-matched healthy individuals, and the information on patients with OA is shown in Supplementary Table 1. We first performed histological assays to evaluate GPR81 expression in knee articular cartilage, examined lactate levels in synovial fluid using an L-lactate assay kit and conducted 16S rRNA sequencing on faecal samples to analyse changes in the gut microbiota. Compared with healthy controls (Ctrl), patients with OA exhibited significant alterations in gut microbiota composition across multiple taxonomic levels. At the phylum level, patients with OA were characterized by a marked enrichment of Firmicutes and Bacteroidetes (Fig. 10a), a hallmark of gut microbial dysregulation^48^. At the genus level, the relative abundance of beneficial taxa, including *Akkermansia*, was notably decreased in patients with OA, whereas proinflammatory genera such as *Veillonella* and *Prevotella* were markedly enriched (Fig. 10b). Consistent with these shifts, class-level analysis revealed a greater abundance of *Clostridia* (within Firmicutes) in the OA group, as well as a reduction in Verrucomicrobiae abundance (Supplementary Fig. 10a). At the order and family levels, these changes were further reflected by diminished proportions of Verrucomicrobiales/Verrucomicrobiaceae, along with an expansion of taxa associated with inflammatory pathways (Supplementary Fig. 10b, c). In our previous data, the abundance of *Akkermansia* increased after MICT intervention, indicating that MICT may regulate OA progression by affecting the abundance of *Akkermansia* in the gut. In addition, correlation analysis revealed that the abundance of *Akkermansia* in faeces correlated negatively with lactate levels in synovial fluid (Fig. 10c). These findings suggest that the reduced abundance of *Akkermansia*, a well-known beneficial bacterium, in OA patients may be a potential contributing factor to the pathogenesis of this disease. The results of histological staining were consistent with those of the animal experiments, which revealed that GPR81 expression was significantly higher in damaged cartilage than in intact cartilage (Fig. 10d, e). These findings indicate that compared with normal cells, damaged chondrocytes are more sensitive to lactate, increasing their sensitivity to lactate regulation and thereby creating a vicious cycle that exacerbates the progression of OA. In addition, the abundance of *Akkermansia* correlated negatively with the expression of GPR81 in cartilage (Fig. 10f), and the expression of GPR81 in cartilage correlated positively with the lactate level in synovial fluid (Fig. 10g). These clinical data demonstrate the relationships among gut *Akkermansia*, synovial fluid lactate and GPR81 expression, reflecting the key role of the gut microbiota–lactate–GPR81 signalling axis in the regulation of OA.

## Discussion

Exercise has been widely reported to be an effective non-pharmacological intervention for OA, but the modulatory effects of exercise at different intensities on OA and the underlying mechanisms remain unclear. In our study, we found that MICT, but not HICT, could effectively alleviate OA. Mechanistically, MICT remodelled the gut microbial composition in DMM mice, which in turn decreased blood lactate levels, further alleviating DMM-induced excess lactate production and Gpr81 overactivation, ultimately ameliorating OA through the Gαi-CREB/NF-κB signalling pathway. Although we did not observe that HICT exacerbated OA progression in our study, we did confirm that MICT could alleviate OA by decreasing lactate levels and purported that inhibiting Gpr81 expression may be a method for OA treatment. Many reports have reported that Metrnl expression can also be increased after high-intensity interval training^10,49^, and these factors have been shown to have beneficial effects on OA under high-intensity interval training intervention, which may counteract the detrimental effects of lactate induced by HICT. Accordingly, no significant difference in OA progression was detected following HICT intervention. Our study highlights the role of OA-associated gut microbiota in lactate metabolism and demonstrates that MICT effectively decreases elevated lactate levels in DMM mice by remodelling the gut microbiome.

Our data demonstrated that MICT was effective at alleviating OA and that ABX treatment significantly attenuated the ameliorative effect of MICT on OA, suggesting that the gut microbiota plays a role in the regulation of OA. LPS is a unique glycolipid and surface antigenic substance in the cell wall of Gram-negative bacteria, and the level of LPS correlates positively with the severity of osteochondritis dissecans and the number of activated macrophages in the synovium^50^. Our results revealed that both the MICT intervention and the ABX intervention reduced the serum concentrations of IL-1β and LPS in DMM mice. Notably, gut microbiota depletion did not abrogate the MICT-mediated reduction in the levels of these proinflammatory markers. These findings indicate that the regulatory effects of MICT on inflammatory factors in DMM mice are independent of the gut microbiota.

Elevated faecal lactate concentrations are commonly observed in a subset of patients with severe ulcerative colitis and Crohn’s disease. However, the potential of lactate-producing bacteria to alleviate the pathogenesis of inflammatory bowel disease (IBD) associated with lactate accumulation remains controversial^51^. Another study demonstrated that lactate-utilizing bacteria effectively ameliorated the pathology of DSS-induced colitis by inhibiting dysbiosis linked to the intestinal inflammatory process and increasing the ratio of *Escherichia coli–Shigella* in the gut. Previous studies have suggested that the main groups of bacteria associated with lactate anabolism are *Lactobacillus*, *Bifidobacterium* and *Lactococcus*^52–54^. We first revealed a class of flora highly enriched in DMM mice after 8 weeks of MICT by constructing a clustered heatmap. The abundance of this class of flora was subsequently linked to lactate, inflammation marker, MMP13 and COL2A1 levels, and the results revealed significant correlations. Notably, we found that *Akkermansia* was the most obvious class of microbes that corresponded to MICT. Although *Akkermansia* has been reported to exert anticancer effects by markedly reducing lactate levels in the tumour microenvironment^55^, our study further verified that this bacterium also alleviates OA by restoring chondrocyte metabolic homeostasis.

Previous studies have suggested that lactate is normally anti-inflammatory and that it negatively regulates the TLR-induced production of NLRP3 inflammatory vesicles and IL-1β via ARRB2 and Gpr81 (ref.^46^). However, lactate appears to be a risk factor for patients with OA, and direct evidence concerning whether the lactate receptor Gpr81 is directly involved in regulating OA is lacking. Our findings also provide direct evidence that Gpr81 is a critical mediator of the effects of lactate on cartilage metabolism. Furthermore, our clinical analyses revealed markedly upregulated Gpr81 expression in the articular cartilage of patients with OA; thus, inhibiting Gpr81 may serve as a novel therapeutic approach to treating OA.

This study has several limitations. Although our data indicate that MICT can influence blood lactate levels through the gut microbiota, evidence of the mechanisms underlying the changes in the gut microbiota driven by MICT is limited. Additionally, although some studies have suggested that lactate has anti-inflammatory effects in various inflammation-related diseases, the role of lactate in OA progression indicates that lactate may regulate many different cell functions in the joint cavity, in which the direct impact of lactate on chondrocyte metabolism may be the major factor in regulating the cartilage environment. Third, the downstream signalling pathway of Gpr81 in chondrocytes should be further investigated by RNA sequence analysis and proteomics, and more detailed exploration of the relationship between Gαi and NF-κB should also be performed in future investigations. In summary, the results of this study revealed that the activation of Gpr81 by lactate promotes articular cartilage damage, manifested as enhanced cartilage degradation and reduced extracellular matrix generation via the Gpr81–Gαi–CREB–NF-κB signalling cascade, which aggravates the pathological process of OA. MICT can decrease high lactate levels in DMM mice through the remodelling of gut microbes, and the Gpr81 inhibitor may be a new strategy for OA treatment.

## Supplementary information

Supplementary Information

## Data Availability

16S rRNA sequencing data in the present study have been deposited in the NCBI Sequence Read Archive database under accession numbers PRJNA1252118.
