# Supplementary material for: The inhibition of lactate/Gpr81 signalling driven by moderate-intensity continuous training protects against osteoarthritis
Source: Exp Mol Med. 2026 Sep 3;58(9):2951–71. doi: 10.1038/s12276-026-01819-8 (PMC13633271; doi:10.1038/s12276-026-01819-8)
Supplement: Supplementary file 1 [file 12276_2026_1819_MOESM1_ESM.pdf]

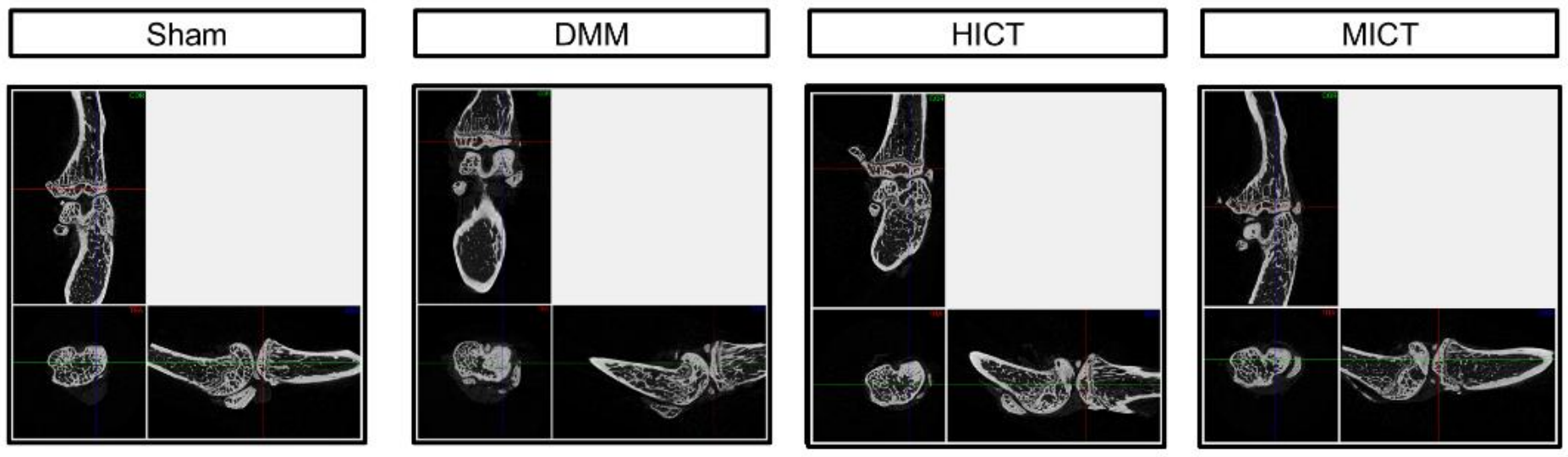

Supplementary Fig. 1 MICT alleviates subchondral bone characteristics in DMM mice. Representative three-dimensional micro-CT images of the knee joints in coronal, horizontal and sagittal sections in Sham, DMM, HICT and MICT groups.

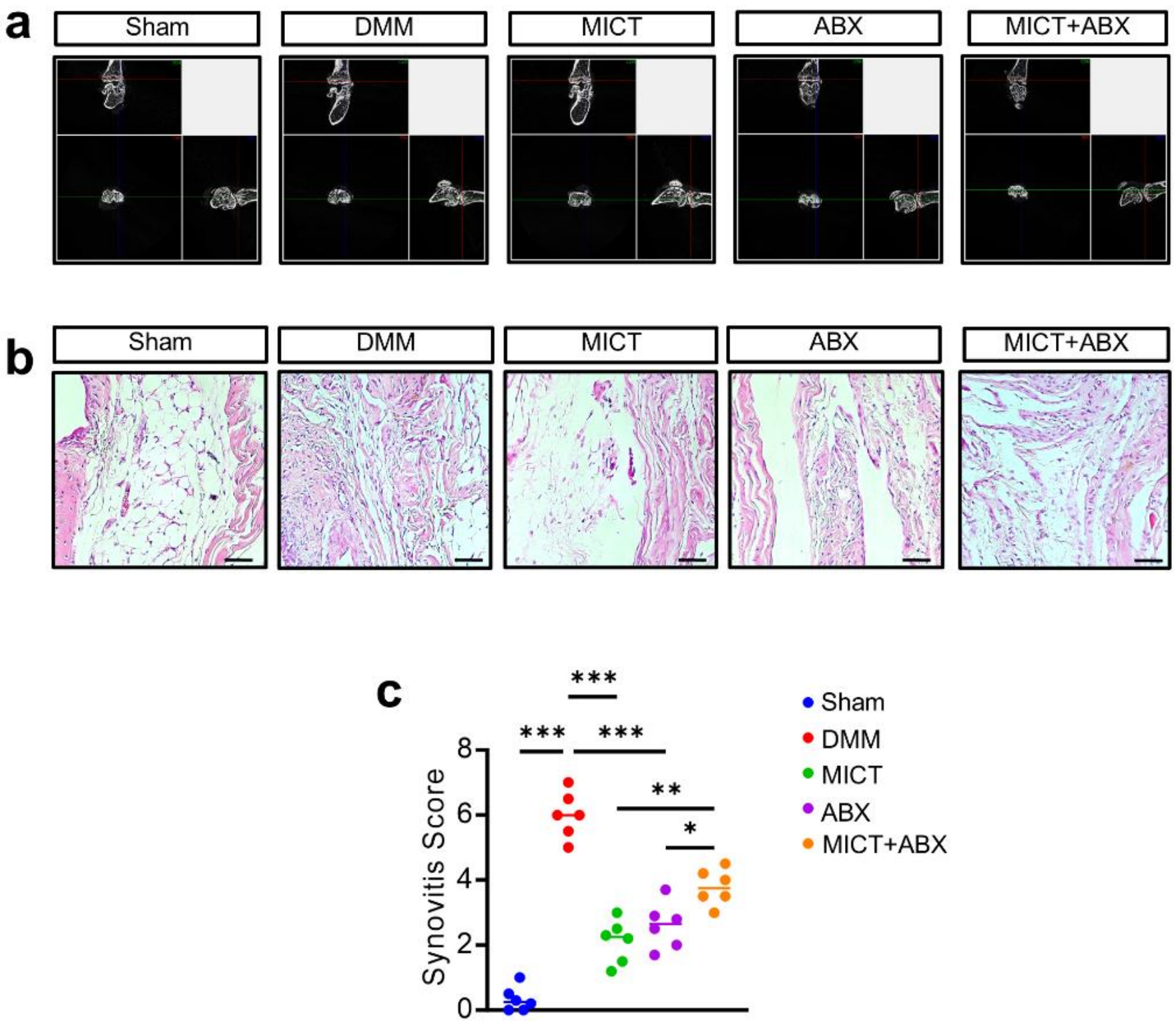

Supplementary Fig. 2 MICT prevents OA via gut microbiota. a Representative three-dimensional micro-CT images of the knee joints in coronal, horizontal and sagittal sections in Sham, DMM, MICT, ABX and MICT+ABX groups. **b** Representative images of H&E staining in Sham, DMM, MICT, ABX and MICT +ABX groups. Scale bar=100 µm. **c** The synovial inflammation score based on H&E staining images of show in (**b**) (*n*=6). * $P < 0.05$, ** $P < 0.01$, *** $P < 0.001$; All data are presented as mean ± SD; Two-way ANOVA analysis followed by Dunnett's post hoc test.

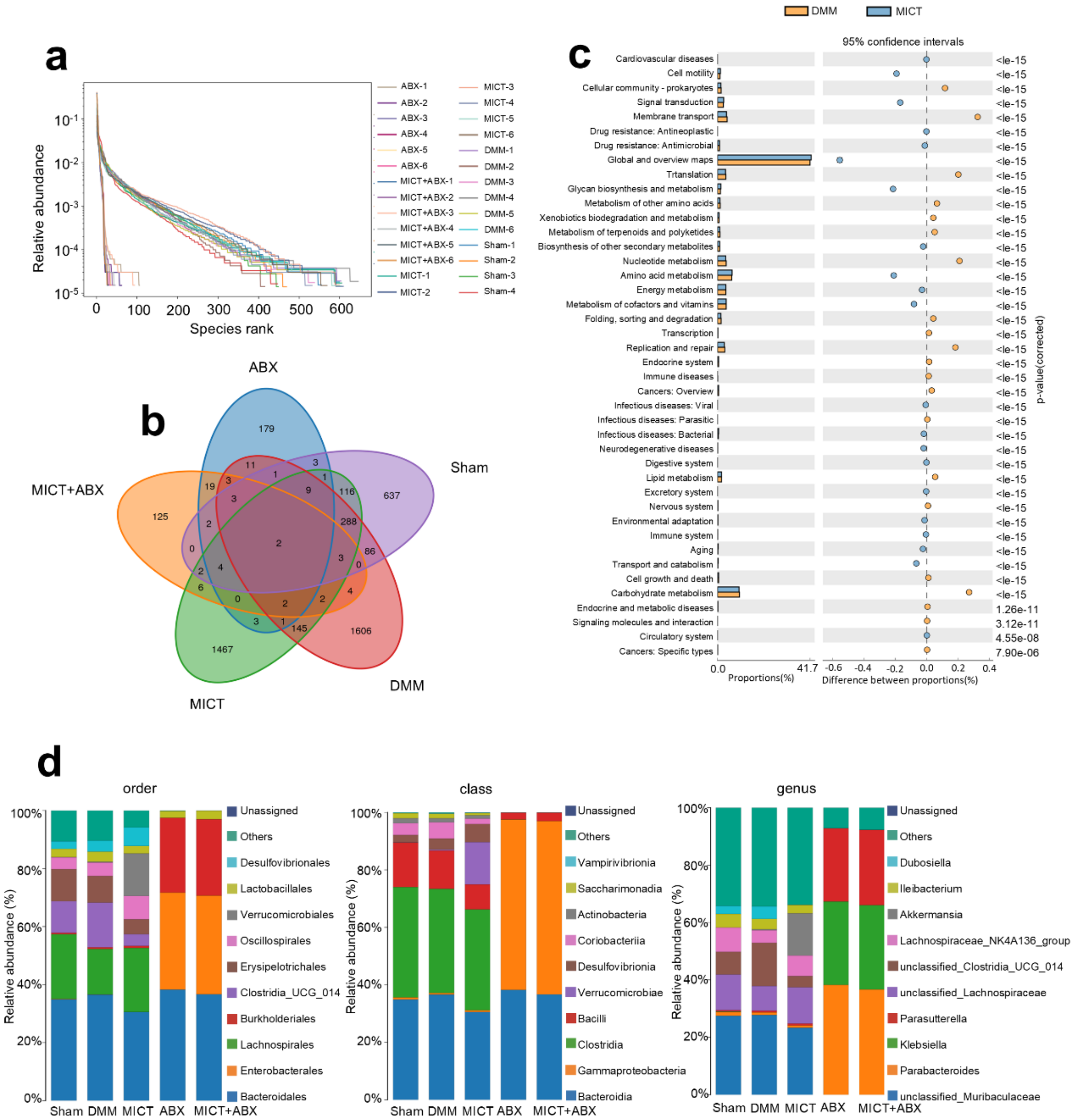

Supplementary Fig. 3 Gut microbial abundance and metabolism were remodeled by MICT.

a The Species rank in the sham, DMM, MICT, ABX and MICT+ABX groups. **b** Venn diagrams of the characteristics of the Sham, DMM, MICT, ABX and MICT+ABX groups. **c** Differential analysis of KEGG metabolic pathways between groups graphs. **d**. Bar chart showing the relative abundance of the top 10 microbiota at the order, class and genus level of cecum content samples in Sham, DMM, MICT, ABX and MICT+ABX groups.

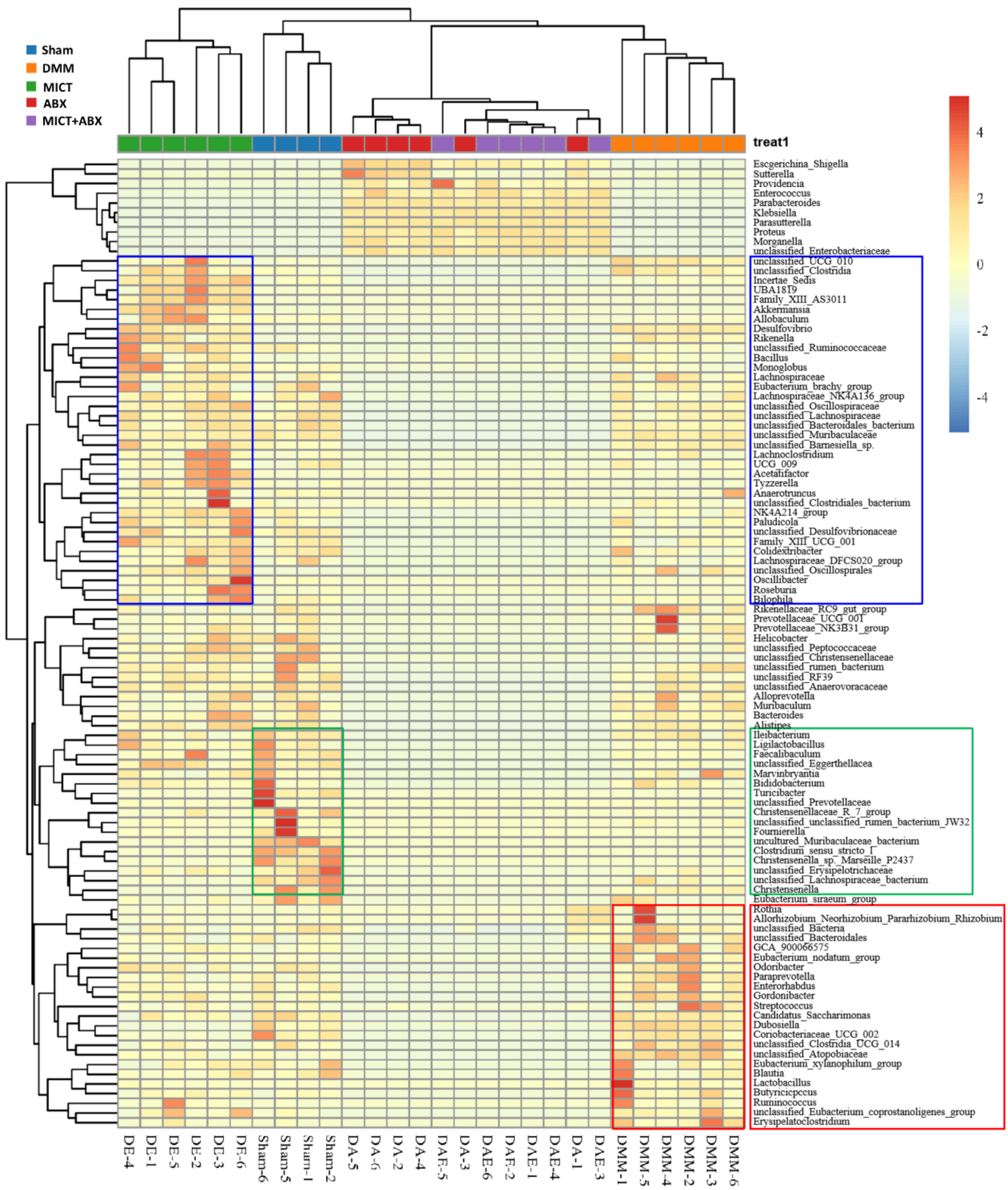

Supplementary Fig. 4 Clustered heat map of relative abundance of gut microbiota in different groups of mice. The blue box represents the microbiota enriched in the MICT groups, the green box represents the microbiota enriched in the Sham groups, and the red box represents the microbiota enriched in the DMM groups.

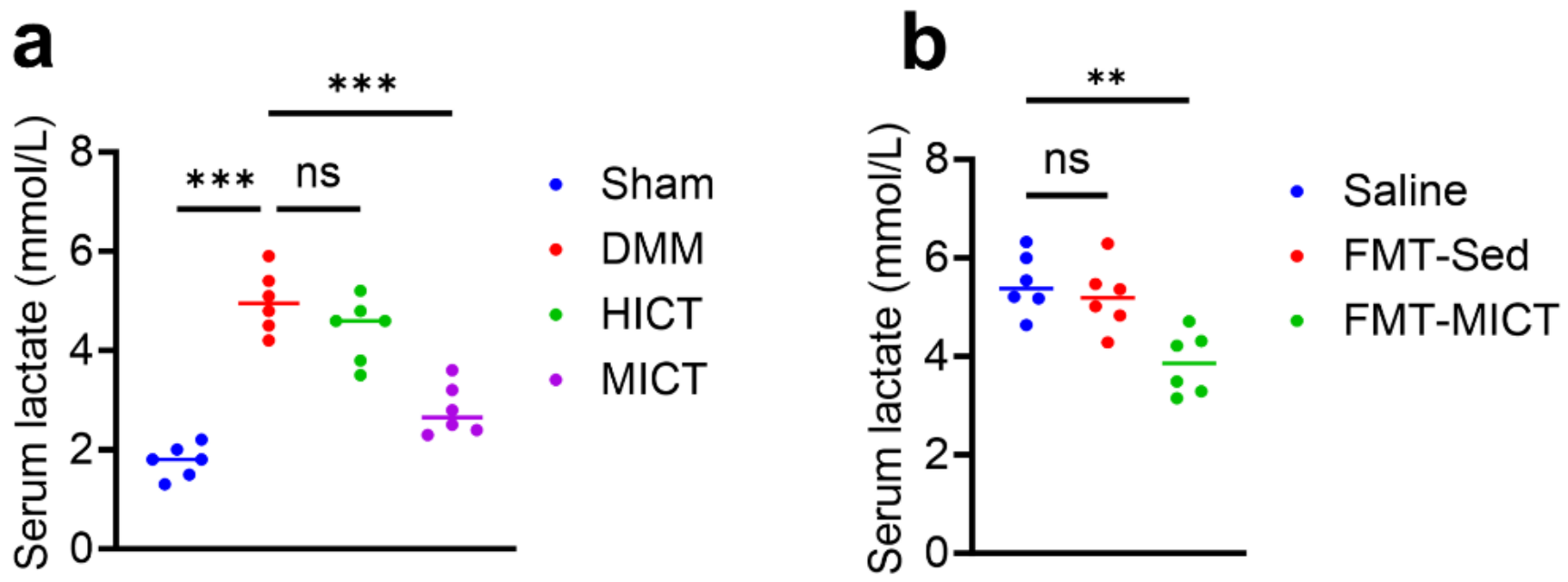

Supplementary Fig. 5 MICT decrease blood lactate by gut microbiota.

a Showing the serum lactate level in Sham, DMM, HICT and MICT groups (n=6). *** $P < 0.001$； ns， no significant. All data are presented as mean ± SD. Two-way ANOVA analysis followed by Dunnett’s post hoc test. **b** Showing the serum lactate level in Saline, FMT-Sed and FMT-MICT groups (n=6). ** $P < 0.01$; ns， no significant. All data are presented as mean ± SD. One-way ANOVA analysis followed by Dunnett’s post hoc test.

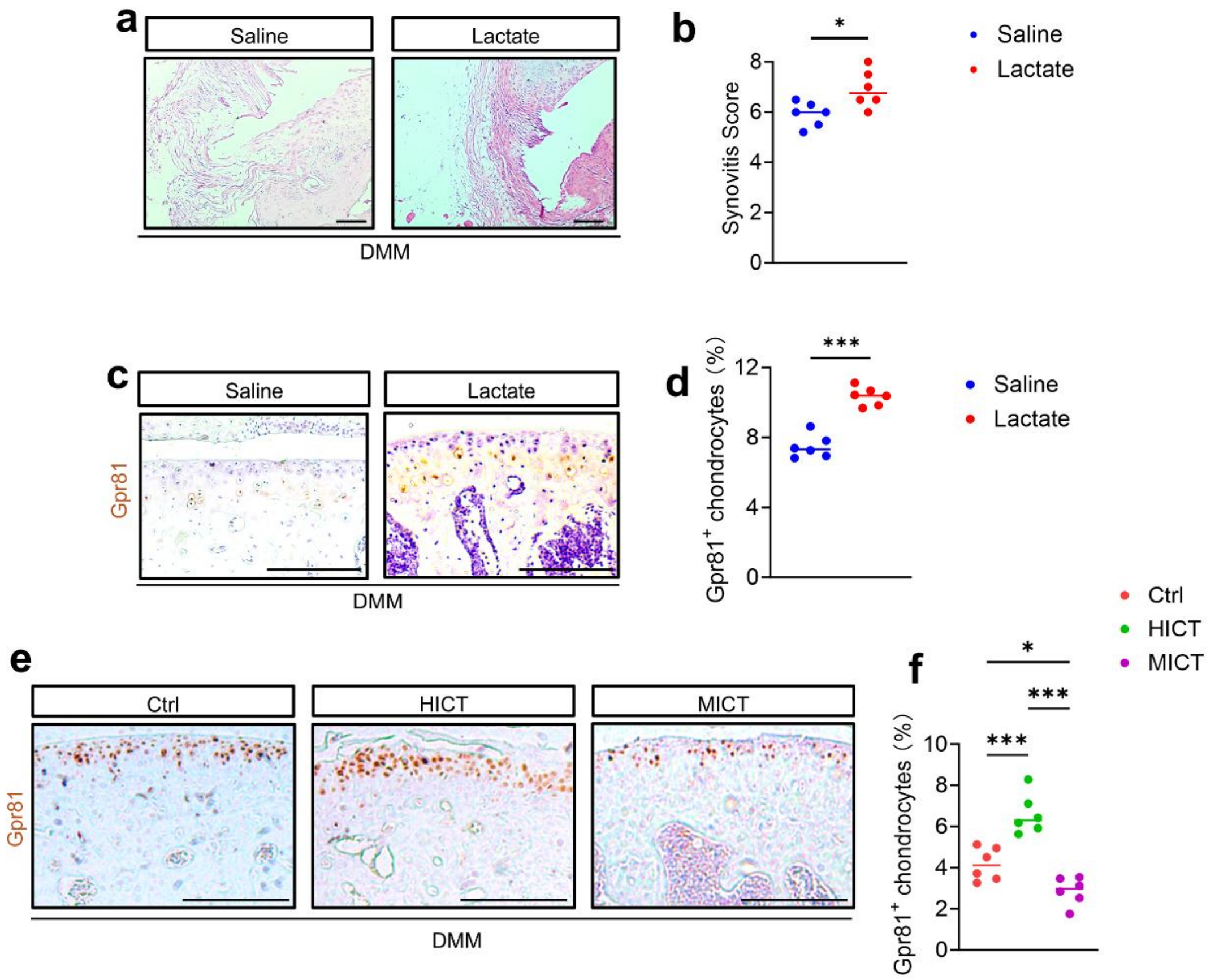

Supplementary Fig. 6 Lactate upregulates Gpr81 expression in chondrocytes *in vivo*. **a** Representative image of H&E staining after Saline and Lactate were injected into the articular cavity. Scale bar=100 μm. **b** The synovial inflammation score based on H&E staining images (n=6). * $P < 0.05$. All data are presented as mean ± SD; Two-tailed Student's t-test was performed. **c** Representative IHC images for Gpr81 expression in articular cartilage of mice after Saline and lactate were injected into the articular cavity. Scale bar=100 μm. **d** Percentage of *Gpr81*$^{+}$ chondrocytes in articular of samples shown in (**c**) (n=6). ***$P < 0.001$. All data are presented as mean ± SD; Two-tailed Student's t-test was performed. **e** Representative IHC images for Gpr81 expression in articular cartilage of mice after HICT and MICT intervention in DMM mice. Scale bar=100 μm. **f** Percentage of Gpr81^{+} chondrocytes in articular of samples shown in (**e**) (n=6). * $P < 0.05$; ***$P < 0.001$. All data are presented as mean ± SD; One-way ANOVA analysis followed by Dunnett's post hoc test.

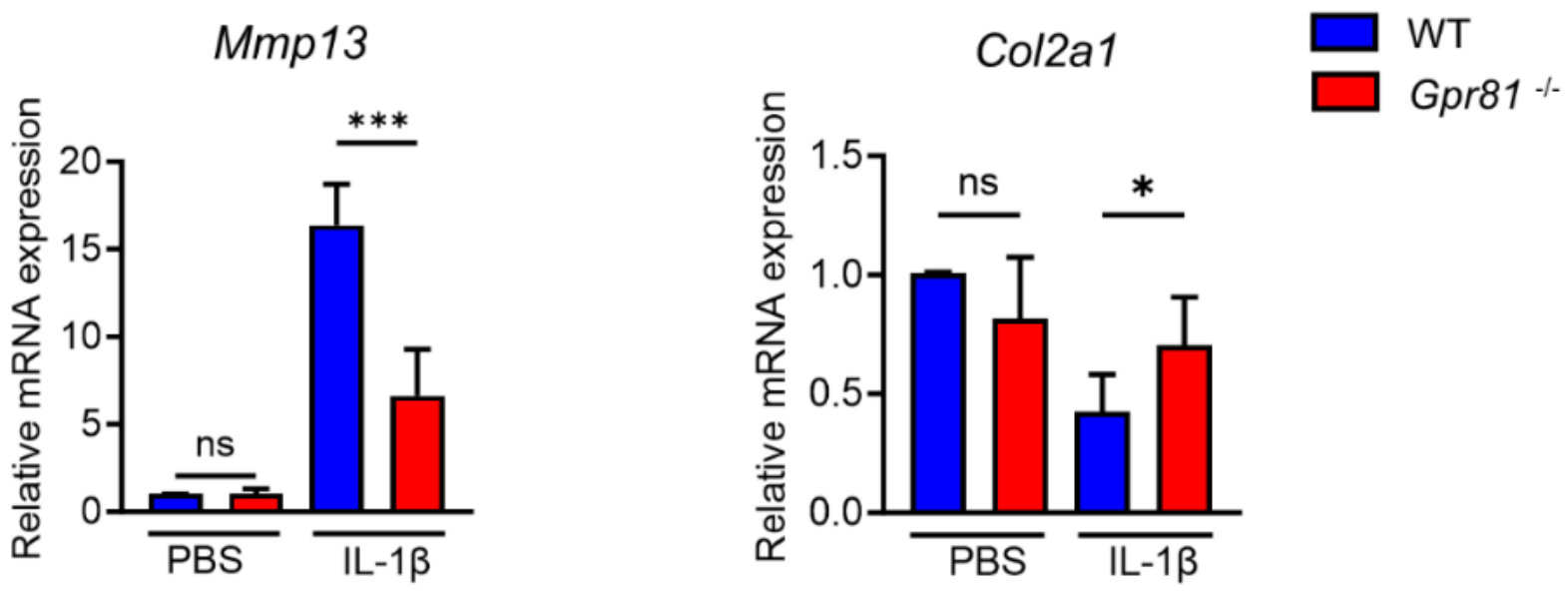

Supplementary Fig. 7 Gpr81 regulates metabolic homeostasis in chondrocytes. BMSC were isolated from wild type and *Gpr81*-deficient mice. The BMSC cultured in the chondrogenic media with IL-1β (1 ng/mL) for 7 days. qRT-PCR analysis of catabolic factors *Mmp13* and anabolic factors *Col2a1* expressing (n=3). * $P < 0.05$, *** $P < 0.001$; ns, no significant. All data are presented as mean ± SD; Two-way ANOVA analysis followed by Dunnett's post hoc test.

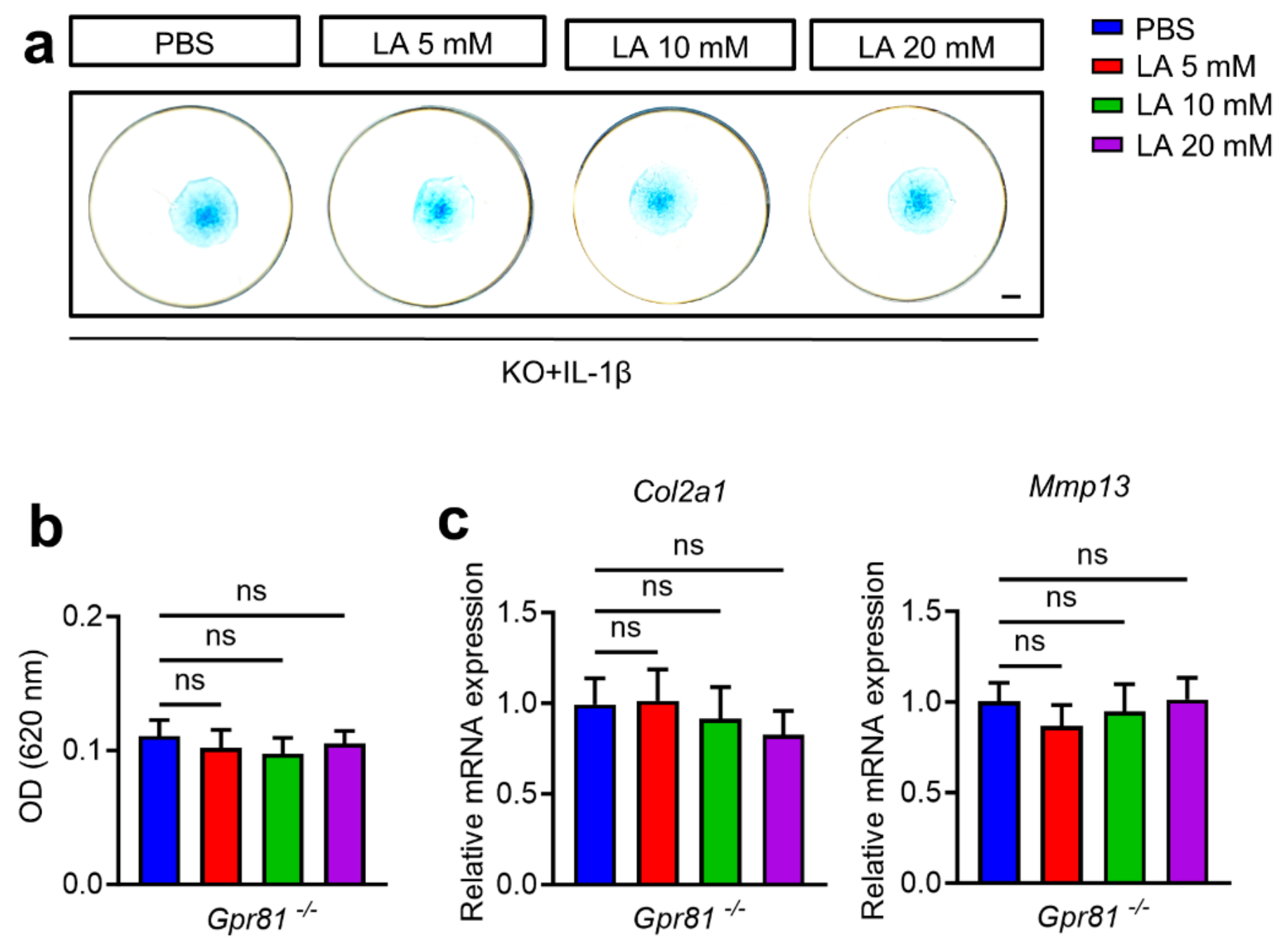

Supplementary Fig. 8 Lactate regulates chondrocyte differentiation via *Gpr81*.

a BMSC were isolated from *Gpr81*-deficient mice. The BMSC cultured in the chondrogenic media with dose dependent lactate for 7 days, then stained with Alcian blue. Scale bar=1 mm. **b** Alcian blue-stained were quantified of show in (**a**) (*n*=3). **c** qRT-PCR analysis of catabolic factors *Mmp13* and anabolic factors *Col2a1* expressed in *Gpr81*-deficient cells treated with dose dependent lactate for 7 days (*n*=3). ns, no significant. All data are presented as mean ± SD; One-way ANOVA analysis followed by Dunnett's post hoc test.

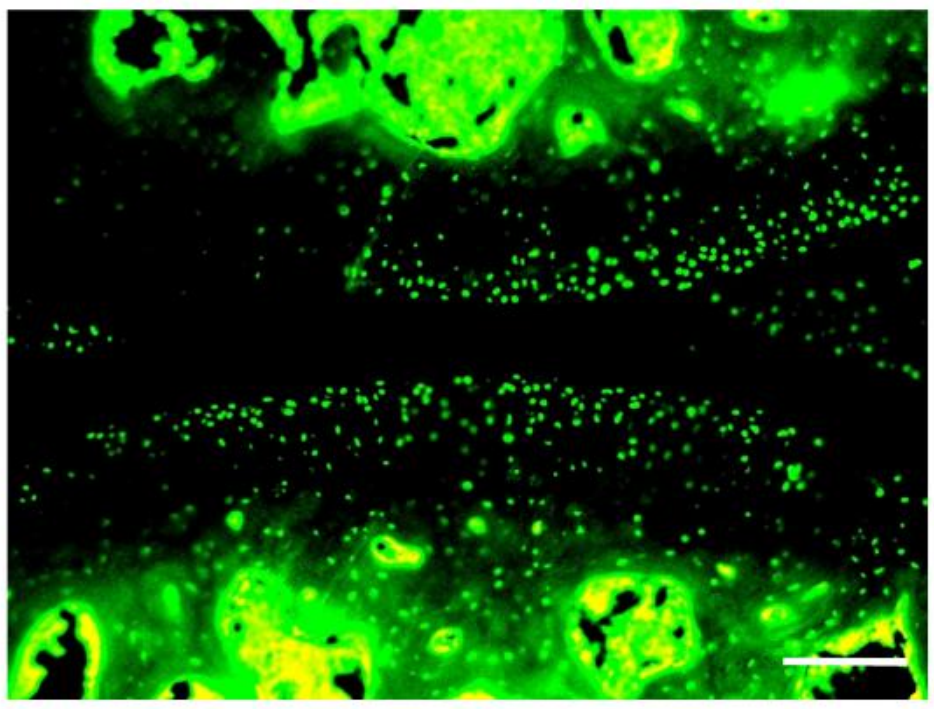

Supplementary Fig. 9 Adeno-Associated Virus serotype 2 (AAV2) represents a great candidate for gene transfer in arthritic cartilage.

Representative immunofluorescence images of adeno-associated virus2 (AAV2) infected articular cartilage for 14 days. Scale bar=100 μm.

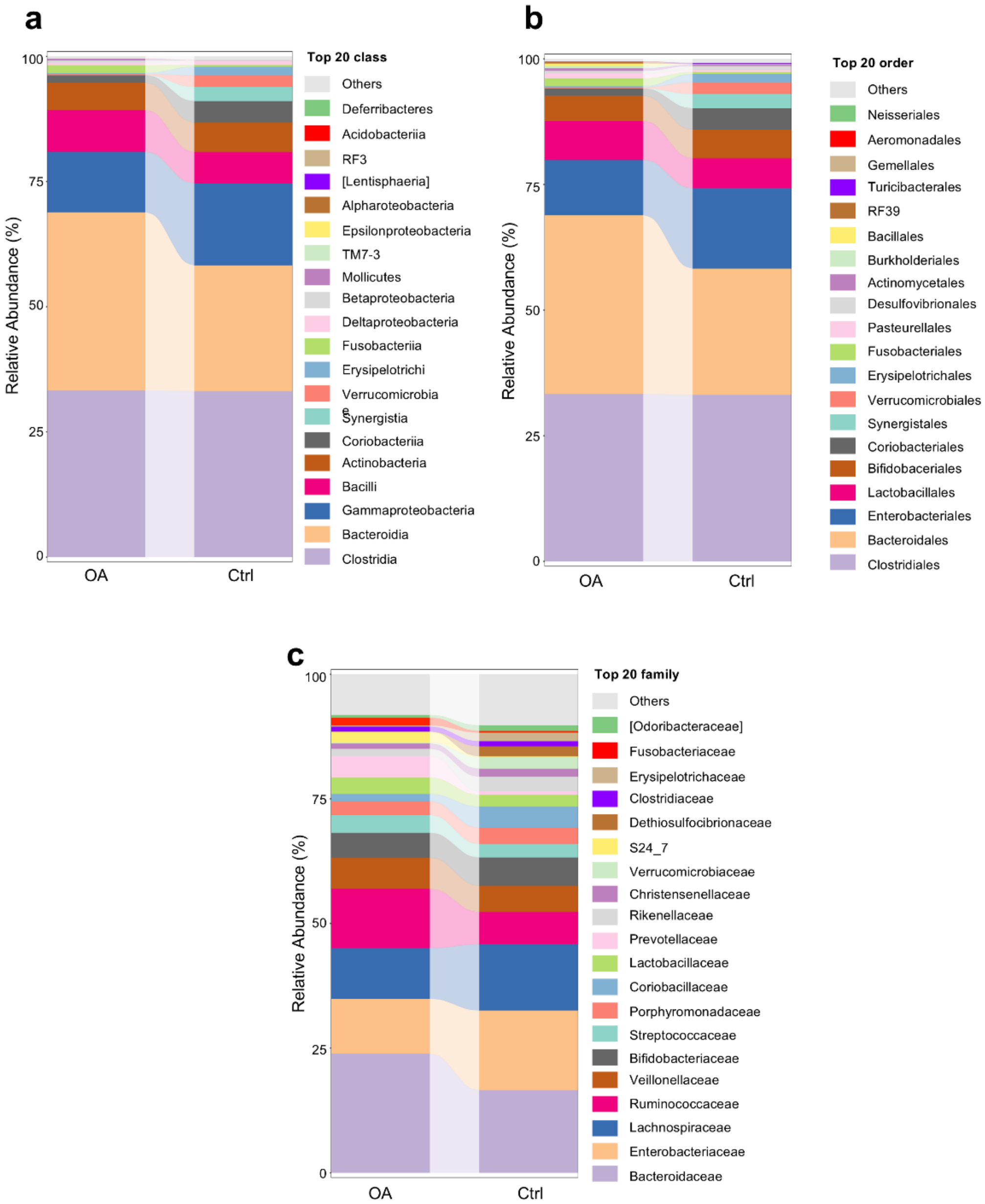

Supplementary Fig. 10 The Composition of the Gut Microbiome in Osteoarthritis patients and healthy individuals.

a Bar chart showing the relative abundance of the top 20 microbiota at the class level in fecal samples from osteoarthritis patients and healthy individuals. **b** Bar chart showing the relative abundance of the top 20 microbiota at the order level in fecal samples from osteoarthritis patients and healthy individuals. **c** Bar chart showing the relative abundance of the top 20 microbiota at the family level in fecal samples from osteoarthritis patients and healthy individuals.

NO.	Age/gender	ICRS Grade	Joint	Use
1	78/F	4	Knee(L)	16s/IHC
2	75/F	3	Knee(L)	16s/IHC
3	80/F	4	Knee(L)	16s/IHC
4	80/F	4	Knee(L)	16s/IHC
5	78/F	3	Knee(R)	16s/IHC
6	80/F	4	Knee(R)	16s/IHC
7	80/F	4	Knee(L)	16s/IHC
8	80/M	4	Knee(R)	16s/IHC
9	79/M	4	Knee(L)	16s/IHC
10	73/M	0	/	16s
11	75/M	0	/	16s
12	78/M	1	/	16s
13	80/M	1	/	16s
14	75/F	0	/	16s
15	73/F	0	/	16s
16	76/F	0	/	16s
17	79/F	1	/	16s
18	80/F	1	/	16s
19	79/F	1	/	16s
20	80/F	1	/	16s
21	75/F	0	/	16s
22	77/F	0	/	16s
23	78/F	0	/	16s
24	78/F	0	/	16s

Supplementary Table 1 Characteristics of specimens from subjects.

ICRS: International Cartilage Repair Society;

L: Left;

R: Right;

IHC: Immunohistochemistry;

M:Male ;

F:Female.
